# Integrated fibre-specific methylome and proteome profiling of human skeletal muscle across males and females with fibre-type deconvolution

**DOI:** 10.1186/s13395-025-00396-0

**Published:** 2025-10-10

**Authors:** Andrew S. Palmer, Esther García-Domínguez, Megan F. Taylor, Andrew Garnham, Kirsten Seale, Joel R. Steele, Han-Chung Lee, Ralf B. Schittenhelm, Nir Eynon

**Affiliations:** 1 Institute for Health and Sport, Victoria University, Footscray, VIC Australia; 2 Australian Regenerative Medicine Institute, Monash University, Clayton, VIC Australia; 3 The Walter and Eliza Hall Institute of Medical Research, Parkville, VIC Australia; 4 Centre for Healthy Ageing, Centenary Institute, Sydney, NSW Australia; 5 Monash Proteomics & Metabolomics Platform, Monash Biomedicine Discovery Institute, Clayton, VIC Australia

**Keywords:** Human-skeletal-muscle, Muscle-fibres, Methylome, Proteome, Deconvolution

## Abstract

**Background:**

Skeletal muscle is an important organ for health and movement, largely driven by specific muscle fibres. However, the comparison of fibre-type-specific DNA methylation and protein abundance from the same sample presents challenges. By combining previous methodological approaches we were able to directly compare the methylome and proteome in Type I and Type II human skeletal muscle fibres in males and females.

**Methods:**

We assessed the methylome using the EPICv2 Infinium array and the proteome using liquid chromatography tandem mass spectrometry (LC-MS/MS) from Type I and Type II fibre pools from both males ($n=7$) and females ($n=5$).

**Results:**

We identified 5,689 robust differentially methylated regions (Fisher *P*-value $< 0.001$) and found strong relationships between methylation and protein abundance in key contractile and metabolic genes. Further, we generated a reference matrix of Type I and Type II fibres and leveraged deconvolution algorithms to accurately estimate fibre-type proportions using whole-muscle DNA methylation data, providing a method to correct for fibre-type in future studies. These results are presented primarily as a resource for others to utilise.

**Conclusion:**

We provide integrated methylome and proteome profiles of human muscle fibre-types generalisable to both male and females as a freely accessible interactive repository, MyoMETH (https://myometh.net), allowing further investigation into fibre regulation. Data are available via ProteomeXchange with identifier PXD066393 and the Gene Expression Omnibus at GSE304045.

**Supplementary Information:**

The online version contains supplementary material available at 10.1186/s13395-025-00396-0.

## Introduction

Skeletal muscle is pivotal for movement, posture and whole-body metabolism, and it’s function is driven by myofibres that can be characterised as slow-twitch (Type I (TI)) and fast-twitch (Type II (TII)) [1]. DNA methylation (DNAm) is an epigenetic modification that influences gene regulation [2] and plays a role in cell-type identity [3]. Changes in skeletal muscle DNAm have been associated with muscle function, health-related phenotypes and healthy ageing [4–10].

Most human studies have analysed whole muscle (WM) samples to investigate alterations in DNAm with limited consideration of fibre-type differences. These studies are constrained by the inability to statistically correct for or study fibre-type differences, making it challenging to rule out the potential confounding effects of underlying fibre-type proportions [11]. Recent work reported transcriptome and proteome differences between human muscle fibres at both the fibre-type and single-fibre level [12–17]. However, the only human study of DNAm in muscle fibres reporting differences between TI and TII muscle fibres was exploratory and limited to sample size of $n=1$ [14]. Other limitations of previous studies on fibre-type specific regulation include the underrepresentation of females and the isolated study of the omic layers.

To overcome these constraints, we aimed to simultaneously extract DNA and proteins from both female and male pooled muscle fibres, to: 1) generate DNAm profiles of whole-muscle (WM), TI and TII muscle fibres; and 2) study the relationship between fibre-type specific DNAm and protein abundance from the same fibres, and 3) compare WM DNAm signatures to those of TI and TII fibre-types to understand the influence of fibre-type in DNAm studies in skeletal muscle.

Here, we present: 1) a robust method to simultaneously extract DNA and protein from the same muscle fibre; 2) a comprehensive DNA methylation analysis of over 800,000 CpGs using the new EPICv2 Infinium array and proteomic profiles of 1,226 proteins using liquid chromatography tandem mass spectrometry (LC-MS/MS) from over 200 myofibres per sample, in both males ($n=7$) and females ($n=5$), and 3) a novel DNAm reference matrix for TI and TII fibres leveraging EpiDISH reference-based deconvolution [18] to infer fibre-type proportions of samples using whole muscle DNAm data. We have generated this data as a resource for further investigation. This resource and the fibre-type estimation tool (MyoTYPE) can be accessed via the interactive web interface, namely MyoMETH (https://myometh.net).

## Methods

### Ethics approval

This study was approved by the Human Ethics and Research Committee at Victoria University, Melbourne Australia (HRE13-223, HRE21-122). Participants were fully informed of the experimental procedure and all associated risks before written and informed consent was obtained from all participants.

### Participants and exclusion criteria

The participants were recruited as part of the ongoing Skeletal Muscle Adaptive Response to Training (Gene SMART) study [19]. Fifteen healthy, non-smoking, males ($n=8$) and females ($n=7$), aged 18-45, volunteered to participate in the study. Medical history was obtained during the first visit and participants were excluded if they had a history of the following medical conditions: coronary heart disease, significant or chronic respiratory conditions, major musculoskeletal problems, uncontrolled endocrine or metabolic disorders or diabetes requiring insulin. Furthermore, individuals were also excluded if they were: females taking hormonal contraceptives, currently pregnant or breast feeding. Muscle biopsies used in this paper were obtained prior to a 12-week exercise intervention.

### Controlled diet

Each participant was provided with individualised, pre-packaged meals for the 48 hours prior to their muscle biopsies. The nutrient needs of participants were calculated using the Mifflin St-Jeor equation [20] and each participant’s weight, height, age and activity level. FoodWorks 10 software (Xyris, Brisbane, Australia) was used to determine the nutritional composition of the prepared meals and ensure the 48-hour diet met participants’ needs, based on the current NHMRC guidelines (50-55% carbohydrates, 15-20% protein, $< 30$% total fat) [21].

### Collection and preservation of muscle tissue

Muscle biopsies were collected, after an overnight fast, from the *vastus lateralis* of the participants dominant leg between 6-8 am under local anaesthesia (3 mL, 1% Lidocaine) by an experienced Medical Doctor using a Bergstrom needle [22]. For female participants muscle biopsies were taken during the first seven days of their menstrual cycle. Biopsied muscle was placed on ice in a glass petri-dish lined with filter paper. Approximately 50-70mg of muscle was immediately snap frozen in liquid nitrogen (stored at $-80^{\circ }$C), approximately 25-40mg of sample was placed in ice cold PBS for single fibre separations, and approximately 10-20mg was embedded in Tissue-Tek O.C.T Compound and frozen on thawing 2-methylbutane.

### Preparation and preservation of skeletal muscle fibres for single fibre isolation

Immediately after the biopsy, muscle stored in ice cold PBS was separated into smaller bundles under a light microscope. Two thirds of the sample was moved to ice-cold RNAlater in a 1.5mL microcentrifuge tube and stored at $-20^{\circ }$C.

### Single fibre isolation, muscle digestion and lysis

We were able to isolate single fibres for 12 out of 15 participants. For single muscle fibre isolation, the RNAlater sample was removed from $-20^{\circ }$C and stored on ice. A ceramic tray on dry ice was prepared and bundles of muscle fibres were isolated in a plastic 60 mm petri dish on the tray with Dumont biological grade tweezers (Dumont, Jura, Switzerland) using a light microscope. Small bundles were transferred to ice cold RNAlater and the remaining muscle was returned to storage at $-20^{\circ }$C. Single fibres were isolated using Dumont biological grade tweezers under the microscope and transferred to 8-strip PCR tubes. After isolation of approximately 24 fibres, 10$\upmu$L of digestion buffer (100 mM TRIS-HCl pH=7.6, 50 mM DTT and 4% SDS) was added to each tube. PCR tubes were spun down for one minute on a tabletop micro-centrifuge and then vortexed for one minute on a MSA vortexer. Tubes were briefly spun again and transferred to $-80^{\circ }$C for storage.

### Dot-blot fibre typing

#### Dot-blot microfiltration

Before fibre-typing, muscle fibres were vortexed for 15 minutes on a vortexer (MSA) at room temperature (RT) to aid in complete digestion of the fibres. A dot blot procedure of muscle fibres as previously described [23] was modified to allow high-throughput and accurate dot blotting. 40-50$\upmu$L of TBS 1X was added to each well of a 96 well plate, along with 2$\upmu$L of each single fibre lysate that had been thawed using a 96-tube vortexer (MSA) at 2500 rpm. The plate was then agitated on an orbital plate shaker for 10 minutes.

A 96 well Bio-Dot Microfiltration Apparatus (BioRad) was assembled with a pre-wet nitrocellulose membrane. 50$\upmu$L of TBS was loaded into each well and filtered through by vacuum suction. 40-50$\upmu$L of protein from the 96 wells were loaded from the agitated plate to the wells of the dotblot apparatus and allowed to filter through completely by gravity for approximately 45-60 mins, then 50$\upmu$L of TBS was added to wash the remainder of the lysate onto the membrane and removed via vacuum suction. The Bio-Dot Microfiltration apparatus was disassembled, and the nitrocellulose membrane was briefly washed in TBS 1X and then allowed to air-dry. The membrane was washed in TBS-T (0.1% Tween20) and blocked in TBS-T with 5% milk for 15 minutes at RT.

#### Antibody incubation and imaging

Membranes were washed 3 x 5 min in TBS-T and incubated overnight with MYH7 antibody A4:840 (RRID: AB_528384, Development Studies Hybridoma Bank (DSHB), Iowa, United States) made fresh every second day at 1:75 in TBS-T with 1% bovine serum albumin (BSA). Membranes were washed 3 x 10 min in TBS-T and incubated with secondary antibody for anti-mouse IgM 1:10,000 (RRID: AB_228329, Thermofisher, Massachusetts, United States) in TBS-T with 5% milk for 1 hour at RT. Membranes were washed 3 x 10 min in TBST-T and exposed to a chemiluminescent substrate for 45 seconds (BioRad, California, United States). The membranes were imaged with a ChemiDoc MP system (BioRad, California, United States) at both high and low-resolution settings for between 30-120 seconds.

#### Stripping and re-probing

Membranes were washed briefly in TBS-T, stripped for 5-10 minutes in PIERCE Restore PLUS stripping buffer (Thermofisher, Massachusetts, United States) at RT, and briefly washed in TBS. The membranes were then re-incubated in secondary antibody for more than 15 minutes and re-imaged to confirm successful stripping. Membranes were then incubated overnight with antibodies for MYH2 antibody A4:74 (RRID: AB_528383, DSHB, Iowa, United States) made fresh every two-three days at 1:200 in TBS-T with 1% BSA. Membranes were prepared as above with secondary antibody for anti-mouse IgG 1:10,000 (RRID: AB_10960845, Thermofisher, Massachusetts, United States). After imaging for MYH2, membranes were washed in TBS, stained with Ponceau red for 10 minutes, rinsed with water to remove excess stain, and imaged using a ChemiDoc MP system.

### Fibre type sample pooling

PCR strip tubes were allowed to thaw by vortexing for 5 minutes at 2,500 RPM on an MSA vortexer at RT. For each sample, TI (MYH7+/MYH2-), TII (MYH7-/MYH2+) and TIIx (MYH2-/MYH7-*ponceau+*) fibres were pooled by transferring 2$\upmu$L of the fibre fragment from the strip tube to a 1.5mL microcentrifuge tube. 100 $\upmu$L of each pooled protein sample was stored at $-80^{\circ }$C for proteomic sample preparation. To the remaining 6$\upmu$L of the sample in the strip tubes, 6$\upmu$L of stabilisation buffer (10mM TRIS-HCl pH = 8.0, 26mM EDTA) was added, briefly vortexed and centrifuged in preparation for DNA extraction. After DNA stablisation buffer was added, 12$\upmu$L of sample from TI, TII or TIIx single fibres were pooled in 1.5mL microcentrifuge tubes. 25$\upmu$g of proteinase K was added and the tube was incubated at $56^{\circ }$C for 4 hours at 1000 rpm in a tube agitator. The numbers of fibres pooled per sample are provided in Supplementary Table S1.

### Pooled fibre type DNA extractions

The following protocol was adapted to extract DNA [24]. Briefly, 200$\upmu$L of 3M sodium acetate (non-adjusted pH) was added to each pooled sample and vortexed for one minute. Next 200$\upmu$L of cool phenol chloroform isoamyl alcohol (PCI) was added to each sample and vortexed for 20 seconds followed by centrifugation for 10 minutes at 18000 x *g* at $4^{\circ }$C. The upper aqueous phase was carefully removed and transferred to a new 1.5mL microcentrifuge tube without disturbing the organic phase. Subsequently, 500$\upmu$L of cool isopropanol was added and mixed by inversion before allowing the gDNA to precipitate overnight at $4^{\circ }$C.

The following day samples were centrifuged at 20000 x *g* for 30 minutes at $4^{\circ }$C, and the supernatant was carefully removed without disturbing the gDNA pellet. The pellets were washed with 100$\upmu$L 70% ethanol and centrifuged at 20000 x *g* for 20 minutes at $4^{\circ }$C. The supernatant was removed and the pellets allowed to air dry at $37^{\circ }$C on a heating block. gDNA pellets were dissolved in TE buffer, re-suspended by gently pipetting up and down and further dissolved by placement on a heating block at $37^{\circ }$C for 30-60 minutes. gDNA concentration was quantified using a Qubit High Sensitivity 1x DNA kit on a Qubit Fluorometer 4 (Thermofisher, Massachusetts, United States) following kit instructions. gDNA A260/280 values were measured on a Nanodrop One (Thermofisher, Massachusetts, United States) unless they contained less than 250ng DNA concentration on the Qubit reading.

### Whole muscle DNA extractions

We isolated gDNA from all 15 participants including the 12 for which we generated pooled fibre type samples. gDNA was extracted from 10-15 mg whole muscle samples homogenised two times at 30 Hz for 30 seconds with a Qiagen TissueLyser II (Qiagen, Netherlands) in 600$\upmu$L RLT Plus Buffer with beta-mercaptoethanol from the AllPrep DNA/RNA/miRNA Universal Kit (Qiagen, Venlo, Netherlands). The kit instructions were followed and gDNA was re-suspended in 50$\upmu$L of AE buffer. The extracted gDNA was passed twice over the column to increase gDNA yield.

### Agarose gel electrophoresis

gDNA samples were visualised on a 1.5% TAE agarose gel prepared with 1x SYBR Safe DNA Gel Stain (Invitrogen, California, United States). 50-75ng gDNA was diluted 1:6 in gel loading dye (Invitrogen, California, United States) and 12$\upmu$L was loaded onto the gel, along with with 2-3 $\upmu$L of 1 Kb Plus DNA Ladder (Invitrogen, California, United States) in one lane. The gel was run at 80 V for 45 minutes and images were taken on a Chemidoc Imaging System (BioRad, California, United States).

### Bisulfite conversion and DNA methylation analyses

For methylation analysis, gDNA samples were distributed using a stratified randomised approach to minimise batch effects, which are prevalent when balanced and randomised approaches are used [25]. Bisulfite conversion and DNA methylation was completed by the Human Genomics Facility of the Genetic Laboratory of the Department of Internal Medicine (HuGe-F) at Erasmus MC (Erasmus, Netherlands). Between 200-250ng gDNA was bisulfite converted using the EZ-96DNA Methylation™MagPrep (ZYMO Research, California, United States) according to the manufacturer’s instructions. The Illumina Infinium MethylationEPIC v2.0 Kit (Illumina, California, United States) was used for DNA methylation analysis. After elution of converted DNA the samples were hybridised to the BeadChip and arrays were scanned using an Illumina iScan system with iScan Control Software v4.0.0.147 and standard image extraction parameters (Illumina, California, United States).

### Power calculations

Using the power calculations from Mansell and others [26], 85% of DMPs showing a mean difference of 10% would be detected at a power of 80%, with a sample size of $n=60$, that is $n=10$ males, $n=10$ females (for 3 fibre types). We were unable to obtain this number of samples due to time constraints and the inability to generate Type IIx samples.

### DNAm data preprocessing and quality control

All processing and analysis were performed using the R statistical computing platform [27] version 4.4.2. Raw IDAT files were processed using the minfi package [28] version 1.50.0. We performed a number of quality control steps, checking that samples had $< 0.01$ mean detection *P* values, and log median intensities of the methylated vs unmethylated channels >10.5. All samples passed a check for potential sex swaps, where the sex of samples were predicted based on the difference in log2 median intensity for the X and Y chromosome probes as implemented in the wateRmelon R package [29] version 2.11.4. Probes with a detection *p*-value $>0.01$, and/or bead count $< 3$ were removed, along with all non-CpG probes, probes located in the sex chromosomes and SNP-related probes. Probes flagged by Illumina as inaccurate or underperforming and cross reactive EPICv1 [30] and 450 K [31] probes were removed. Furthermore, off-target probes were removed and replicate probes were averaged using the remap functions in DMRcate [32, 33]. Normalisation of type I and type II probes were performed using the Dasen normalisation method [29] as implemented in the wateRmelon R package [29] version 2.11.4. Principal component regression was utilised to identify biological and technical sources of variation in DNAm using the pcrplot function in the ENmix package [34] version 1.40.2.

### Identifying differentially methylated positions and differentially methylated regions

We identified DMPs using the limma [35, 36] package version 3.60.6 in R by calculating empirical bayes moderated t-statistics. The model was grouped according to fibre-type/tissue (TI, TII, WM). Whilst sex was included as a co-variate, ethnicity was not due to low ethnic diversity in sampling. Duplicate correlation was added to control for the clustering of samples by participant due to the paired experimental design. All samples collected as part of the broader fibre type methylome and proteome project were included in the analysis. We identified robust differentially methylated regions (DMR), defined as regions with $\ge$ 4 CpGs using the DMRcate package [33] version 2.17.0 in R. Differential methylation analysis was conducted on the more statistically valid M-values, however beta value changes were also produced by running the models with beta values. To correct for multiple testing in the identification of DMPs the Benjamini and Hochberg (BH) FDR method [37] was used.

### Gene set enrichment analysis of DMPs and DMRs

Over representation analysis (ORA) of DMPs and DMRs was conducted using the MissMethyl package [38] version 1.38.0 which was modified for compatibility with the EPICv2 to adjust for bias caused by the differing numbers of CpGs mapping to each gene. GO and Reactome pathways were used in ORA and all genesets with $< 5$ or $>500$ genes were removed prior to analysis.

### Deconvolution of whole muscle DNAm data

To generate reference matrix profiles for TI and TII fibres, we took the top 10, 20, 50, 100, 200 and 1000 CpGs in equal portions (i.e. half hypermethylated half hypomethylated) after ranking using a combination of beta logFC x adjusted *p*-value. The reference matrix was used with the EpiDISH package [18] version 2.20.1 in R using the RPC method.

### Proteomics sample preparation

Protein lysates (100$\upmu$L) were prepared from human muscle fibre samples ($n=50$) using a buffer containing 5% SDS and 100 mM Tris-HCl, pH 8.0. The lysates were heated to $95^{\circ }$C for 10 minutes to denature proteins and deactivate enzymes. DNA was sheared by sonication using a Qsonica Q125 ultrasonic processor (Qsonica, Connecticut, United States). Sonication was performed in three 30-second pulses with cooling on wet ice between cycles. Insoluble debris was removed by centrifugation at 14,000 $\times$ g for 10 minutes. The supernatant was processed using the S-trap sample preparation protocol as described by Zougman and others [39]. Proteins were digested overnight at $37^{\circ }$C using trypsin (Trypsin Gold, Promega, Wisconsin, United States) at an enzyme-to-protein ratio of 1:50. After digestion, peptides were acidified with 1% trifluoroacetic acid and purified using Stage-tips packed with SDB-RPS discs, following the protocol described by Kulak and others [40]. iRT peptides (Biognosys) were added to all samples to enable retention time normalization during liquid chromatography tandem mass spectrometry (LC-MS/MS) analysis. Peptide concentrations were determined using a NanoDrop A205 spectrophotometer (NanoDrop One, Thermo Fisher Scientific, Massachusetts, United States). The injection volumes for LC-MS/MS analysis were adjusted to ensure that 250 ng of peptides were loaded on-column for each run.

### Liquid chromatography tandem mass spectrometry

LC-MS/MS analysis was conducted using a Dionex Ultimate 3000 RSLCnano system (Thermo Scientific) coupled to an Orbitrap Q-Exactive HF mass spectrometer (Thermo Scientific). Peptides were loaded onto an Acclaim PepMap RSLC C18 analytical column (75 $\upmu$m $\times$ 50 cm, 2 $\upmu$m particle size, 100Å pore size) and separated at a flow rate of 300 nL/min. Prior to separation, peptides were trapped on a PepMap 100 C18 trap column (100 $\upmu$m $\times$ 2 cm, 5 $\upmu$m particle size, 100Å pore size). Chromatographic separation was achieved using a linear gradient of Buffer A (0.1% formic acid in water) and Buffer B (80% acetonitrile with 0.1% formic acid), starting with 5% Buffer B and increasing to 35% over 90 minutes of linear separation. Mass spectrometric data acquisition was performed in data-dependent acquisition mode. Full MS scans were collected at 60,000 resolution (m/z 200), and MS/MS scans were acquired at 15,000 resolution using higher-energy collision dissociation (HCD). Fragmentation was performed with a normalized collision energy of 28%. For each duty cycle, a single full MS1 scan (120,000 resolution at m/z 200; AGC $3\times 10^6$; max IT 54 ms; 375–1575 m/z) was followed by up to 12 dd-MS$^2$ scans (Top12) acquired at 30,000 resolution (AGC $2\times 10^5$; max IT 54 ms; 200–2000 m/z). Precursors were isolated with a 1.4 m/z window (fixed first mass 120 m/z), dynamic exclusion was 15 s, and isotopes were excluded.

### Inclusion list generation

Proteotypic peptides for inclusion in the targeted analysis were generated using the Picky application https://picky.mdc-berlin.de/. The inclusion list was curated to target myosin isoforms associated with muscle fibre types. The database used for generating the inclusion list is based on ProteomeTools, as described by Zolg and others [41] and further implemented in the Picky spectrum library by Zauber and others [42]. An inclusion list of 40 isoform-unique MYH peptides (MYH1/2/4/7) was generated from in-house DDA discovery runs as well as utilization of the PickyDB. Criteria included uniqueness to the target isoform, tryptic peptides without missed cleavages, length 8–20 amino acids, and predicted high ionisation efficiency. The list was embedded in all DDA runs to bias precursor selection (not PRM). For these targeted events we used the same dd-MS$^2$ settings as above (resolution 30,000; AGC $2\times 10^5$; max IT 54 ms; NCE 27). The complete list (m/z, charge, peptide sequence, protein accession) is provided in Supplementary Table S2.

### Proteomics data analysis

Raw data were analyzed using FragPipe version 19.1 with the MSFragger version 3.5 search engine [43]. Protein identifications were made using the Human SwissProt proteome (accessed June 2020) with inclusion of common contaminants and iRT standards. The search parameters included carbamidomethylation of cysteine residues as a fixed modification and oxidation of methionine and N-terminal acetylation as variable modifications. The false discovery rate (FDR) for peptide-spectrum matches and protein identifications was set to 1%, validated using Percolator. Label-free quantification was performed using IonQuant version 1.10.27 [44]. Data analysis was performed in R version 4.4.2. Proteins identified as contaminants, decoy sequences, or detected in fewer than three replicates were excluded from further analysis. Label-free quantification (MAXLFQ) intensities were log2-transformed and normalized using a median-centering approach. Missing values were imputed assuming a missing-not-at-random pattern, with global imputation performed by drawing random values from a left-shifted Gaussian distribution (1.8 standard deviations, width 0.3).

### Myosin isoform analysis

Individual myosin composition was determined exclusively from peptides that map unambiguously to a single isoform. The Unique Intensity values reported by IonQuant in the protein.tsv output were extracted for MYH1, MYH2, MYH4 and MYH7. For each sample, unique intensities were first summed within each isoform to obtain a protein-specific intensity. The four isoform intensities were then summed to a grand total, and isoform abundance was expressed as a percentage of this total:

$$\begin{aligned} \% \text {MYH}_i = \frac{\sum _{j=1}^{n} \text {Unique Intensity}_{ij}}{\sum _{k=\{1,2,4,7\}} \sum _{j=1}^{n_k} \text {Unique Intensity}_{kj}} \times 100 \end{aligned}$$

where i denotes the isoform and j indexes its unique peptides. Peptides shared between isoforms (razor or non-unique) were excluded to avoid cross-assignment [45, 46]. All calculations were performed in R 4.4.2 and the analysis script is provided in the code repository.

### Identifying DEPs

Differentially expressed proteins (DEPs) were identified using the limma package version 3.60.6 in R [36]. Empirical Bayes moderated t-statistics were applied to protein-wise linear models. Duplicate correlations were included in the model to account for the paired experimental design. Fibre type, defined by the expression of MYH7 (Type I) or MYH2 (Type IIa), was used as the primary grouping factor. Sex was included as a covariate in the model. Statistical significance was determined using an adjusted *p*-value threshold of 0.001, corrected for multiple testing using the Benjamini-Hochberg method [37], and a log2 fold-change threshold of $\mid 1 \mid$.

### Gene set enrichment analysis of DEPs

ClusterProfiler [47, 48] version 4.12.6 was used to conduct ORA of DEPs in GO, KEGG and Reactome gene sets, excluding those gene sets with $< 5$ or $>500$ genes. We used the 1226 proteins used in the differential protein analysis as a background list.

## Results

### Method for simultaneous extraction of DNA and proteins and fibre typing

#### Simultaneous preparation of DNA and protein enables integrated methylome and proteome profiling

**Fig. 1:**
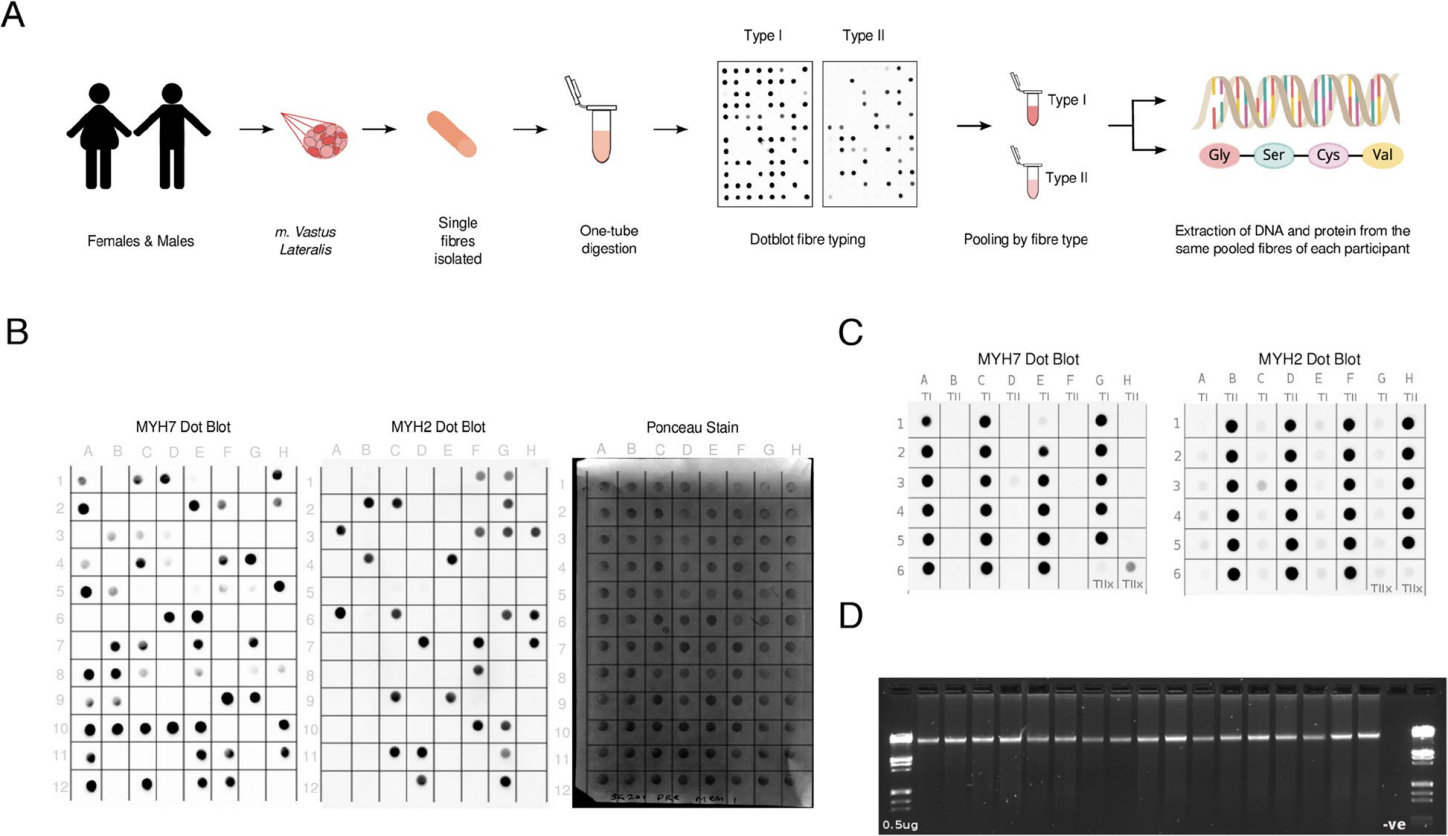
Simultaneous extraction of DNA and proteins and fibre typing. **A** Single muscle fibres were isolated and typed from *vastus lateralis* muscle from males and females and both protein and DNA simulataneously extracted from pooled fibre populations. **B** Representative dot blot of 96 single fibres. Left: MYH7 type I (TI) blots. Middle: MYH2 type II (TII) blots. Right: MYH7-/MYH2- type IIx (TIIx) blots. **C** Pooled sample dot blots. Left: MYH7 TI blots, Right: MYH2 TII blots. **D** Representative agarose gel for pooled fibre type gDNA

We have simultaneously separated proteins and DNA from muscle fibre types, which represent pure populations of TI slow and TII fast fibres as determined by myosin heavy chain (MYH) patterns (Fig. 1A). To achieve this, we isolated $>200$ fibres per individual from the *vastus lateralis* muscle taken from 7 males and 5 females (Supplementary Table S3), which were lysed and typed as either TI (MYH7), TII (MYH2) or TIIx (MYH7-/MYH2-/ponceau+) (Fig. 1B). We pooled one fifth of the single fibre lysates and confirmed that pooled TI lysates were positive for MYH7 and negative for MYH2, whereas pooled TII lysates were positive for MYH2, and showed some amount of MYH7 staining (Fig. 1C). Pooled TIIx lysates had minimal staining for both MYH2 and MYH7 (Fig. 1C). We then performed untargeted quantitative proteomics on these lysates using LC-MS/MS. We extracted gDNA from the remaining three fifths of the pooled lysates. The gDNA was intact (Fig. 1D) with an A260/A280 value of >1.7, and approximately 250ng per sample was subsequently analysed using the EPICv2 Infinium array. In addition, we extracted gDNA from whole muscle tissue from 15 participants (12 with pooled fibre samples) to enable a comparison of DNAm between the fibre types and whole muscle. This modified approach has enabled us to generate both methylome and proteome data from the same fibres for the first time.

### Protein differences between TI and TII fibres

#### LC-MS/MS analysis

**Fig. 2:**
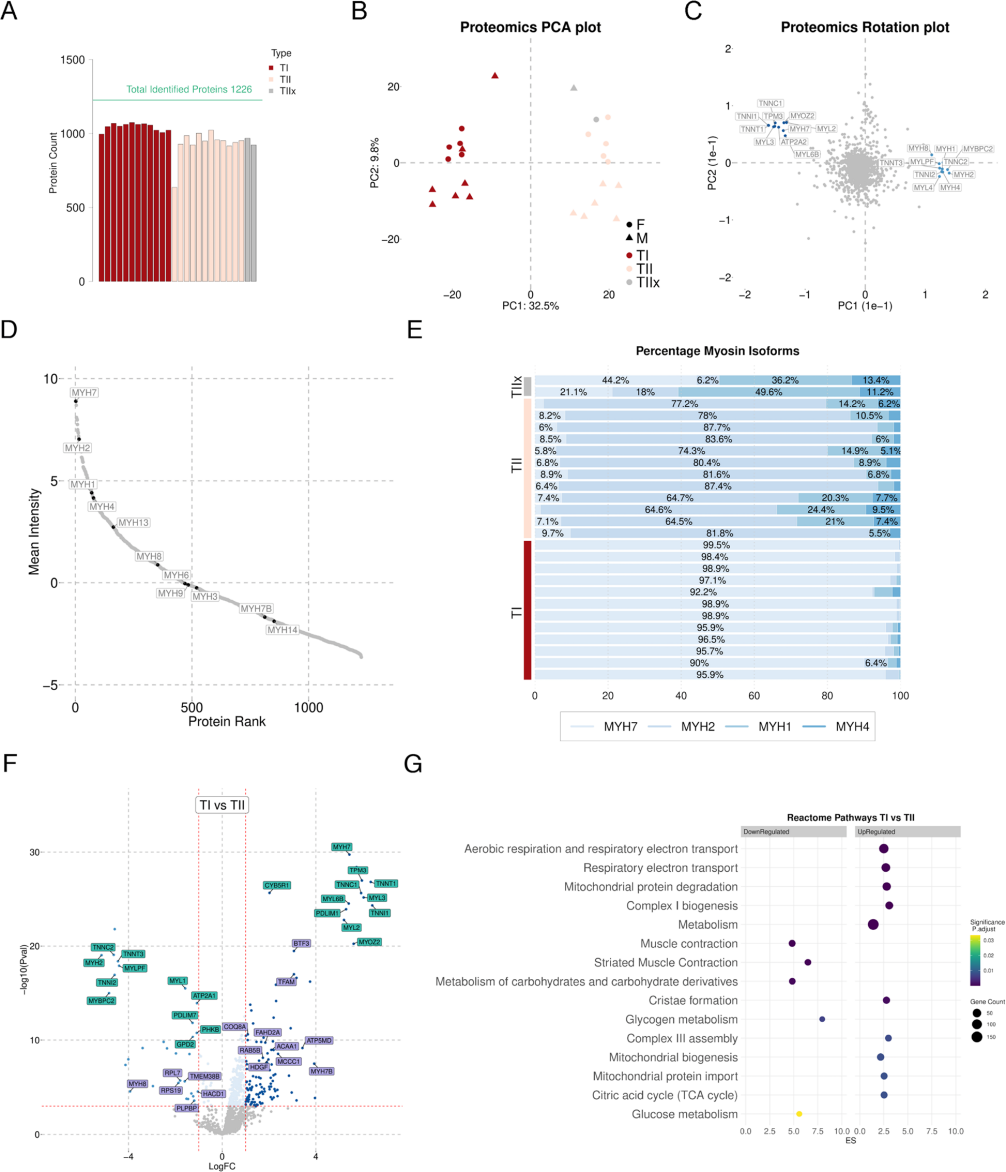
Protein analysis of pooled TI and TII fibres. **A** Number of proteins identified in each sample. A total of 1,226 proteins were identified using LC-MS/MS-based proteomics. **B** PCA of proteome data. Red: TI oxidative fibres, white: TII glycolytic fibres grey: Expected TIIx fibres; circles: males (M); triangles: females (F) (C) PCA loadings of the proteome data showing the top 10 features underlying the separation of samples. **D** Ranked intensities of myosin proteins identified in all samples. **E** Abundance of MYH7, MYH2, MYH1 and MYH4 isoforms in the pooled samples. Abundance calculated as percentage of total MYH7, MYH2, MYH1, MYH4 signal intensity in each sample. Red: TI samples, white: TII samples, grey: TIIx samples. **F** Volcano plot of DEPs identified between type I (TI) and type II (TII) fibres, with logFC on the X-axis and -log10 *P*-value on the Y-axis. Royal blue: down-regulated and logFC $< -1$, dark blue: up-regulated and logFC $>1$, in TI compared with TII fibres. The horizontal line represents the cutoff of the corresponding adjusted *p*-value $< 0.001$ and the vertical lines represent the logFC cutoff of 1 and −1. Proteins labelled in green are the overlaps with previous papers and those in purple were not. **G** Reactome GSEA of both up-regulated and down-regulated DEPs between TI and TII fibres ($p < 0.001$ and logFC $>0$). The circle size represents gene counts and the colour represents the adjusted *p*-value and the enrichment score on the X-axis

We assessed protein abundance differences between TI and TII fibres using untargeted quantitative proteomics and identified a total of 1,226 proteins across the samples after protein filtering. Protein counts were consistent across samples and conditions (mean = 984, SD = 88), except for one TII sample (male), which had a reduced number of identified proteins (636) and was excluded from subsequent analysis (Fig. 2A). To determine the number of proteins belonging to sarcomeric/contractile proteins, we conducted a search in UniProt for all proteins detailed as sarcomere or contraction. 14.3% (175) of the 1,226 proteins overlapped with this list. Principal component analysis (PCA) showed that 32.5% of the variation in the protein data was explained by the first dimension and samples clustered according to TI and TII fibres (Fig. 2B). Analysis of PCA loadings revealed that myosin and troponin isoforms, and ATP2A2 separated the proteome data along dimension 1 (Fig. 2C).

We assessed myosin proteoform content to determine the purity of each pooled sample by analysing unique peptide intensities (Additional file 2). We identified a total of 11 myosin class two proteins in the samples of which MYH7 and MYH2 were found to be highly abundant (Fig. 2D). In alignment with previous studies [15, 16], and the dot blots (Fig. 1C), our data show that MYH7 positive TI fibres express mostly MYH7 (on average 97% MYH7 expression), and MYH2 positive fibres express mostly MYH2 (on average 77% MYH2) (Fig. 2E). Type II fibres show a higher proportion of MYH1 and MYH4 with on average 12% and 5% respectively, in comparison to TI fibres. MYH4 content of muscle fibres can be challenging to measure accurately and is dependent on proteomic method chosen [49]. What we thought to be Type IIx fibres showed a mixed percentage of MYH7, MYH2, MYH4 and MYH1 and are likely not representative of TIIx fibres (Fig. 2E). From this point TIIx fibres have been disregarded and the TII fibre pools are considered TIIa/TIIx samples. The myosin isoform percentages can be found in Additional file 3.

#### Robust protein differences between TI and TII fibres related to contraction and metabolism

A comparison of TI and TII samples revealed 425 and 338 differentially expressed proteins (DEPs) with a Benjamini-Hochberg False Discovery Rate (BH-FDR) $p < 0.005$ and $p < 0.001$, respectively. Of the proteins passing BH-FDR $< 0.001$, 144 DEPs showed a log-fold-change (logFC) of $>1$ or $< -1$ (‘robustDEPs’) (Supplementary Table S4). Among these robustDEPs 110 were up-regulated and 34 were down-regulated in TI fibres compared with TII fibres. In agreement with previous work [15, 17], the top down-regulated proteins were MYH2, TNNC2, TNNI2, TNNT3, MYL4 and MYLPF, and the top up-regulated proteins were ATP2A2, MYH7, TPM3, TNNT1, MYL3 and TNNC1 (Fig. 2F). 54 of our robust DEPs overlapped both previous pooled-fibre analyses and 52 robust DEPs were not previously identified as differentially regulated in these studies (Additional file 4). We show evidence that key mitochondrial proteins TFAM, COQ8A, FAHD2A, ATP5MD, MCCC1 were upregulated in TI compared with TII fibres (Fig. 2F). In contrast, ribosomal proteins RPL7 and RPS19 and the sarcoplasmic reticulum protein TMEM38B were downregulated in TI compared with TII fibres. Consistent with previous observations, we showed that PDLIM1 was up-regulated in TI fibres compared with TII fibres [15]. Furthermore, there was higher expression of PDLIM7, which is reported to be a TIIx specific protein [16], in TII fibres compared with TI fibres, confirming that the TII fibre population is likely a combination of TIIa and TIIx fibres.

To determine pathways and processes that are over-represented in the DEPs we conducted over representation analysis (ORA) using the clusterProfiler [47, 48] package in R. Using the 283 up-regulated DEPs irrespective of any logFC cutoff, we identified 10 enriched Reactome terms with a BH-FDR $p < 0.05$. Using the 56 down-regulated DEPs irrespective of any logFC cutoff, we identified 5 enriched Reactome terms with a BH-FDR $p < 0.05$. There were overlaps between up-regulated DEPs and TCA cycle, respiratory electron transport, mitochondrial protein degradation, complex I biogenesis and metabolism Reactome pathways (Fig. 2G). Down-regulated DEPs were enriched in Reactome pathways related to muscle contraction, carbohydrate metabolism, glucose metabolism and glycogen metabolism (Fig. 2G). We show that our pooled samples are truly representative of TI and TIIa/TIIx fibres and have robust differences in proteins involved in mitochondria and contraction. The proteome analysis results can be found in Additional file 4 and FragPipe output files in Additional files 5-8.

### DNAm differences between TI and TII fibres

#### Methylation analysis

**Fig. 3:**
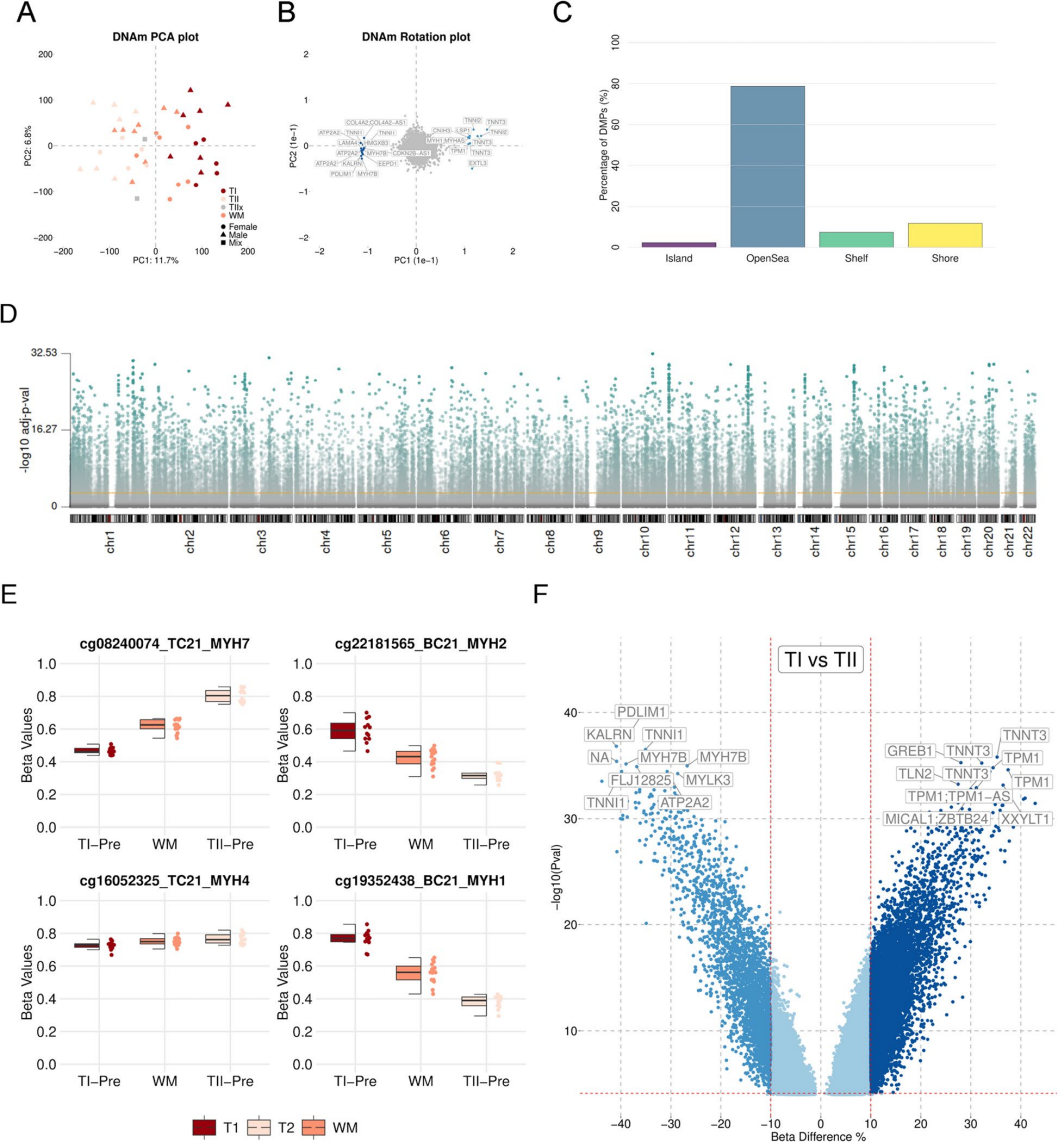
Differentially methylated positions in TI vs TII fibres. **A** Principal component analysis (PCA) of DNAm data. Red: TI oxidative fibres, white: TII glycolytic fibres grey: whole muscle samples; circles: males (M); triangles: females (F); squares: mixed TIIx samples. **B** PCA loadings of the DNAm data showing the top 10 features underlying the separation of samples. **C** Genomic context of DMPs, purple: Islands, blue: OpenSea, green: Shelves, yellow: Shore. **D** Distribution of all CpGs across the autosomes. Y-axis is the -log10-adjusted-*p*-values and the orange line represents the cut of of *p*-values $<0.001$. **E** Boxplots of the top DMP identified with limma in MYH7, MYH2, MYH4 and MYH1. Red: TI fibres ($n=12$), orange: whole muscle (WM) ($n=15$), beige: TII fibres ($n=12$). **F** Volcano plot of DMPs identified between TI and TII fibres, with Beta difference on the X-axis and -log10 *p*-value on the Y-axis. Royal blue: hypomethylated, dark blue: hypermethylated in TI compared with TII fibres. The horizontal line represents the cutoff of the corresponding adjusted *p*-value $<0.001$ and the vertical lines represent the cutoff of the beta difference 10% and −10%

Genome-wide DNAm analysis was performed on 819,560 DNAm sites. A Welch’s t-test revealed a hypermethylation (0.2%) of TI samples compared to TII samples ($p < 0.001$). PCA showed that 11.7% of variation in the methylation data was explained by the first dimension with TI fibres clustering away from TII fibres and whole muscle samples (WM) dispersed through the middle (Fig. 3A). DNAm PCA loadings revealed that myosin and troponin, and *ATP2A2* separated the methylome along dimension 1 (Fig. 3B) in agreement with the proteome data (Fig. 2C), strengthening the evidence that we have generated pooled samples representative of TI and TII fibres.

#### Differentially methylated positions

A comparison of TI and TII samples ($n = 12$ vs 12) revealed 92,007 and 67,049 differentially methylated positions (DMPs) after BH correction at stringent FDR thresholds of $p < 0.005$ and $p < 0.001$, respectively (Supplementary Table S5). Of the 67,049 DMPs passing FDR $< 0.001$, 11,188 DMPs had a beta value difference greater than 10%, which we refer to as ’robustDMPs’ from herein. The robustDMPs were predominantly located in the opensea CpG context with 78.4% located in CpG openseas, 11.8% in CpG shores, 7.4% in CpG shelves and 2.4% in CpG islands (Fig. 3C), which are similar percentages to those previously reported [14]. RobustDMPs were distributed along all autosomal chromosomes (Fig. 3D). Among the robustDMPs 8,408 were hypermethylated and 2,780 were hypomethylated in TI fibres compared with TII fibres (Supplementary Table S5). The top DMP annotated to *MYH7* and *MYH2* showed a distinct methylation difference as previously reported [14] with WM samples in between the two fibre types (Fig. 3E). We also show the same pattern for *MYH1* (Fig. 3E). MYH4 showed reduced differences between fibre types in agreement with the low levels of MYH4 expression in human muscle fibres. In TI compared with TII fibres the top hypomethylated CpGs were annotated to *PDLIM1*, *KALRN*, and *TNNI1* and the top hypermethylated CpGs were annotated to *TNNT3*, *GREB1* and *TPM1* (Fig. 3F). Our methylation data is in strong agreement with our protein data and provides additional evidence that we have successfully generated a robust dataset.

#### Differentially methylated regions

**Fig. 4:**
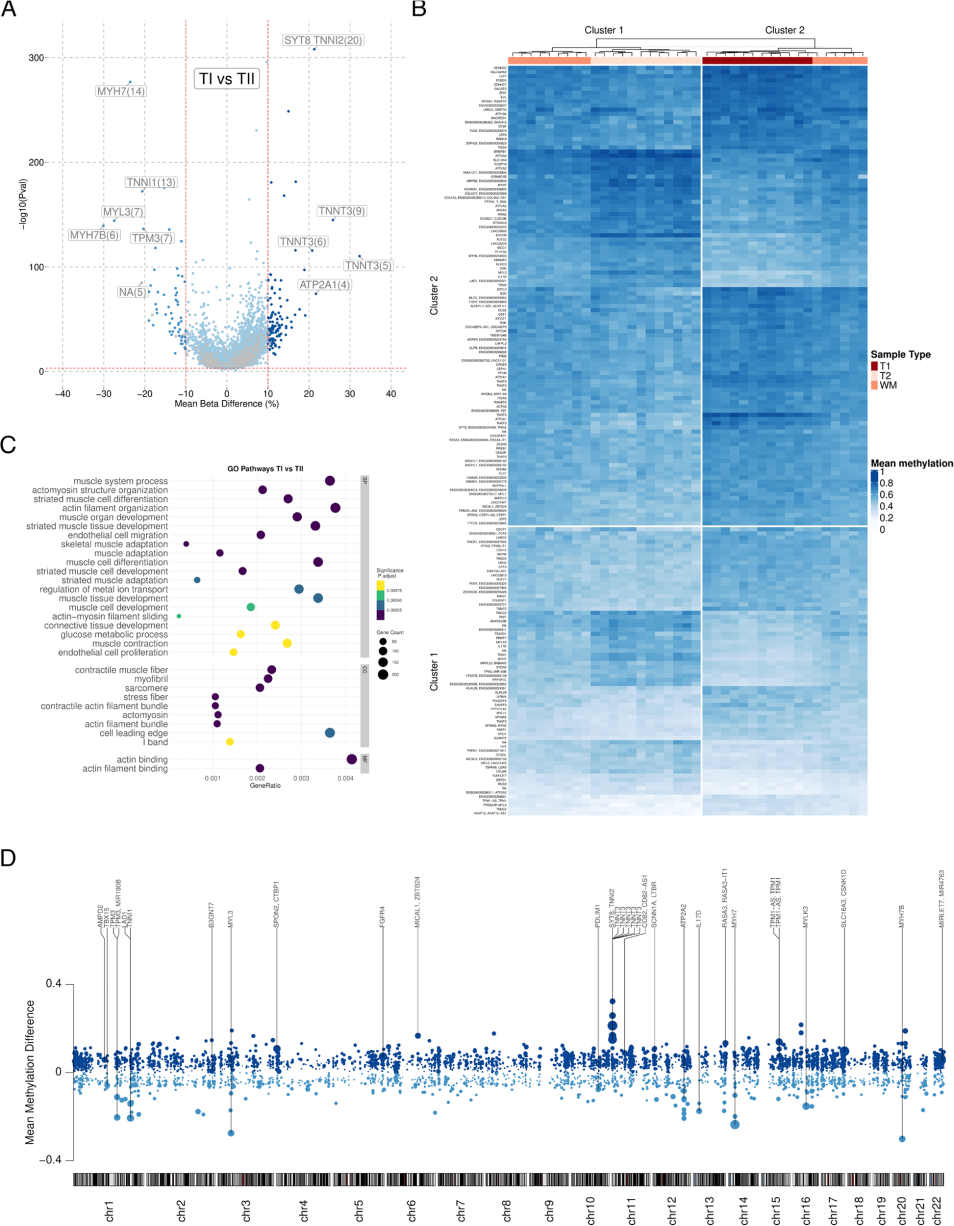
DNAm differences between TI and TII fibres. **A** Volcano plot of the robust DMRs identified between type I (TI) and type II (TII) fibres, with mean beta difference on the X-axis and -log10 *p*-value on the y-axis. Light blue: hypomethylated dark blue: hypermethylated in TI compared with TII fibres. Grey dots are DMRs annotated to no gene, and the number in brackets indicate the number of CpGs in the DMR. The horizontal line represents the cutoff of the corresponding adjusted *p*-value $< 0.001$ and the vertical lines represent the cutoff of the beta difference 10% and −10%. **B** Heatmap of mean methylation levels for the 151 robust DMRs with $>10$% DMR wide mean methylation difference. Red: TI oxidative fibres, white: TII glycolytic fibres, orange: whole muscle samples. **C** Gene ontology (GO) gene set enrichment analysis of the DMRs between TI and TII fibres for: BP: biological processes, CC: cellular component MF: molecular function GO terms. The circle size represents gene counts and the colour represents the adjusted *p*-value and the GeneRatio enrichment on the X-axis. **D** Visual representation of the autosomal location of robust DMRs between TI and TII fibres. Light blue: hypomethylated regions, dark blue: hypermethylated regions. Mean methylation difference between TI and TII fibres are on the Y-axis with genomic location on the X-axis. Size of the dots represent adjusted *p*-value with larger dots representing a smaller adjusted *p*-value

We investigated differentially methylated regions (DMRs) in TI compared with TII muscle fibres using the DMRcate [33] package in R. We identified a total of 8,443 ranges with $>2$ proximal CpGs of which 5,689 ranges had $>3$ proximal CpGs with a Fisher *P*-value $< 0.001$ (robustDMRs) that were annotated to 5,856 unique ensembl genes (one DMR can be annotated to multiple genes). Of these robustDMRs, 4,102 were hypermethylated and 1,587 were hypomethylated in TI compared with TII fibres (Fig. 4A), and 179 had $>10$% mean methylation difference between TI and TII fibres. Using heatmap clustering (K means clustering) on these 179 robustDMRs, we identified two distinct top-level clusters, which clearly separated TI and TII fibres (Fig. 4B). Several genes contained multiple robustDMRs, with *TNNT3*, *TNNC2*, *ATP2A1*, *KLHL29*, and *MAD1L1* identified as hypermethylated and *MYH7*, *IL17D*, *TPM3*, *KIAA1217*, and *ATP2A2* hypomethylated in TI compared with TII muscle fibres. Gene set enrichment analysis (GSEA) of the CpGs from the robustDMRs were over-represented in 32 muscle-related biological process, cellular component and molecular function terms (Fig. 4C) with adjusted *p*-values $< 0.001$. The muscle-related terms were related to muscle cell development and differentiation and contractile filament organisation, suggesting the methylation patterns of myonuclei are involved in the determination of fibre identity. DMRs were present across all autosomal chromosomes and the top DMRs were annotated to key myosin, titin, troponin, and tropomyosin genes (Fig. 4D) involved in skeletal muscle contraction.

Both the DMP and DMR analysis revealed widespread and robust DNAm differences between TI and TII muscle fibres. There were clear methylation differences in contractile genes that are known to be differentially regulated in muscle fibres providing evidence that DNAm plays a role in fibre-type specification. Due to the large numbers of identified differences in DNAm between TI and TII fibres, we have developed a data repository of these results for further enquiry called MyoMETH (https://myometh.net).

### Relationships between methylome and proteome

To assess the link between DNAm status and protein expression in TI and TII muscle fibres, we matched the 1,226 proteins to CpG probes on the EPICv2 array. Of these, 1,143 proteins had at least one CpG site annotated to them. Among the 338 DEPs with a BH-FDR $p < 0.001$, 215 overlapped with at least one DMP. A moderate negative Pearson’s correlation was observed between the beta logFC of the top DMPs for each gene and protein expression, r(1139) = −0.40, $p = < 0.001$ (Fig. S1A). When methylation differences were averaged across the entire gene (mean beta logFC), the reduction in noise resulted in a stronger Pearson’s correlation between differential methylation and protein expression, r(1139) = −0.58, $p = < 0.001$ (Fig. S1B).

We conducted ORA on Reactome pathways and GO-terms to identify the classes of genes over-represented in each of the following overlapping categories; HyperDMP/DownRegulated, HypoDMP/UpRegulated, HyperDMP/UpRegulated and HypoDMP/DownRegulated in a TI vs TII comparison. No over-represented Reactome or GO terms were identified using either the genes/proteins from the HyperDMP/UpRegulated and HypoDMP/DownRegulated overlaps. However, glycogen metabolism, carbohydrate metabolism, glycolysis, glycogen breakdown and muscle contraction were over-represented Reactome terms in the HyperDMP/DownRegulated overlaps. Moreover, muscle contraction and striated muscle contraction Reactome terms were over-represented in the HyperDMP/DownRegulated overlaps (Fig. S1C).

**Fig. 5:**
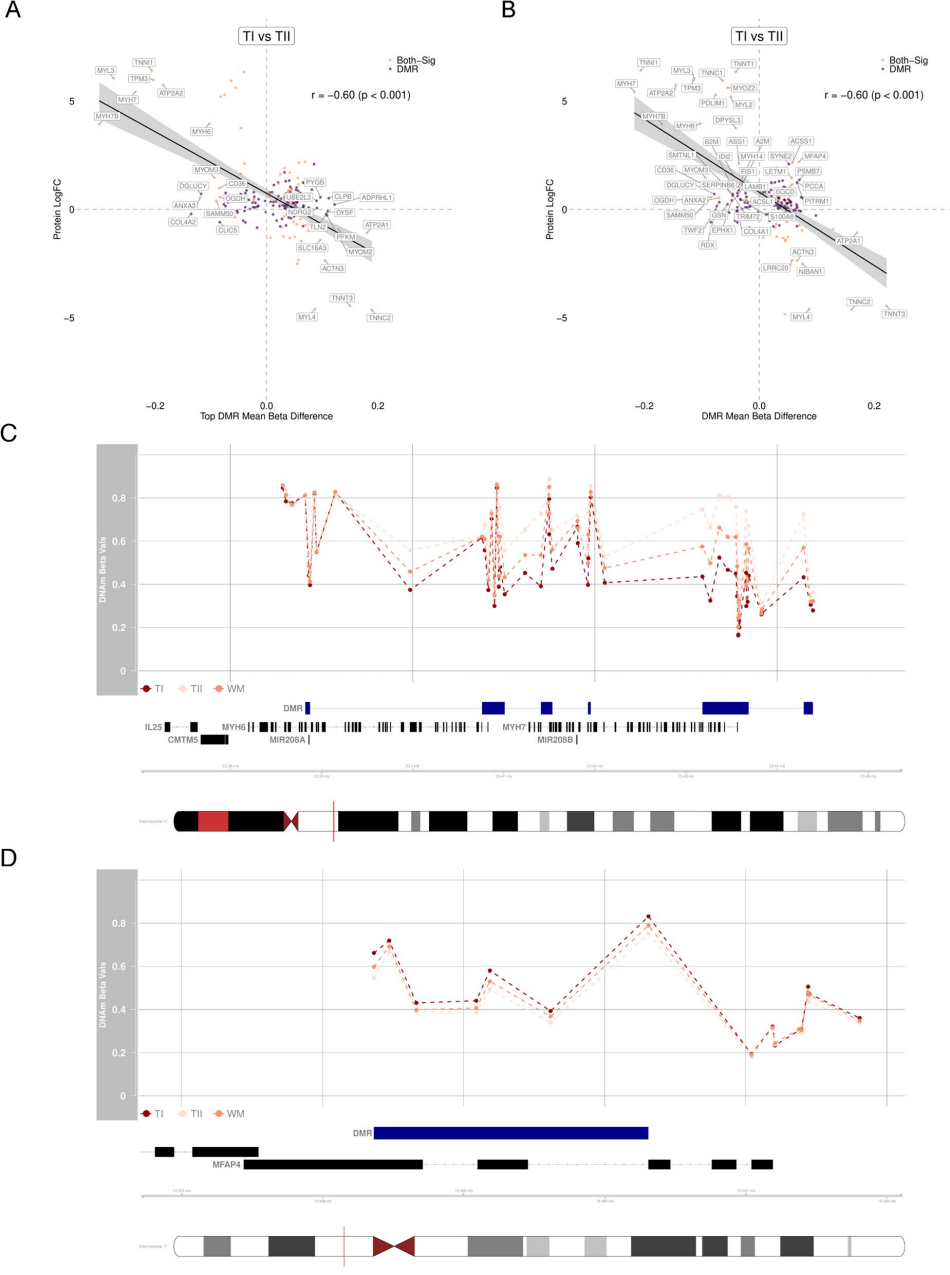
Relationships between methylome and proteome. **A** The Pearson’s correlation between the mean beta difference for the top DMR and protein logFC in each DMR with a measured protein [r(196) = −0.60, $p = < 0.001$]. Purple: only DMRs and orange: both DMR and DEP. The Y-axis is the beta difference between type I(TI) and type II (TII) fibres and the X-axis is the logFC difference of proteins between TI and TII fibres. **B** The Pearson’s correlation between the mean Beta difference for all DMRs and protein logFC in each identified gene with a measured protein [r(196) = −0.60, $p = < 0.001$]. Purple: only DMRs and orange: both DMR and DEP. The Y-axis is the beta difference between TI and TII fibres and the X-axis is the logFC difference of proteins between TI and TII fibres. **C** Visualisation of methylation values across chr14:23,385,520-23,444,000. The genome visualisation shows all the CpGs within this region. DMRs are indicated by blue blocks, the gene models are indicated by black gene tracks. **D** Visualisation of methylation values across chr17:19,382,509-19,388,127. The genome visualisation shows all the CpGs within this region. DMRs are indicated by blue blocks, the gene models are indicated by black gene tracks

We analysed the relationship between the top DMRs (mean methylation difference) and logFC protein expression for 198 proteins that corresponded to a robustDMR. As the methylation values were taken across multiple CpGs, the Pearson correlation between methylation and protein expression increased. A moderate-strong negative Pearson correlation was observed for both topDMR methylation vs logFC protein expression (r(196) = −0.60, $p = < 0.001$), and meanDMR methylation vs logFC protein expression (r(196) = −0.60, $p = < 0.001$ respectively (Fig. 5A, B). The top hypermethylated DMRs between TI and TII fibres were annotated to key contractile genes such as *MYL4*, *TNNC2*, *TNNT3*, which had lower protein expression (Fig. 5A, B). Conversely, the top hypomethylated DMRs in TI vs TII fibres were annotated to key contractile genes such as *MYH7*, *ATP2A2*, *TNNI1* and *MYL3*, and these had higher protein expression levels (Fig. 5A, B).

Most of the DMR/protein overlaps show the expected relationship of either hypermethylation associated with protein down-regulation, or hypomethylation associated with protein up-regulation. For example, the *MYH7* region on chr14 (chr14:23,385,520-23,444,000), which encodes both *MYH7* and *MYH6*, was hypomethylated at transcription start sites (Fig. 5C), and there was up-regulation of protein expression in TI vs TII fibres. Interestingly, several DMR/DEPs show the opposite relationship with hypermethylation and higher protein abundance. For example, *MFAP4* was hypermethylated in the gene body (Fig. 5D) which is a known characteristic of transcribed genes [50]. This was aligned with the up-regulation of protein expression in TI fibres despite a hypermethylation in TI compared to TII fibres.

We report a moderate to strong relationship between methylation and protein differences in TI and TII myofibres, particularly in key genes/proteins involved in skeletal muscle fibre type specification. We also provide evidence that key genes/proteins involved in substrate metabolism and energy systems have higher levels of DNAm and lower levels of protein expression in TI compared to TII skeletal muscle fibres. Methylation analysis results can be found in Additional file 9.

### Whole muscle DNAm signature is strongly influenced by TI and TII fibres

**Fig. 6:**
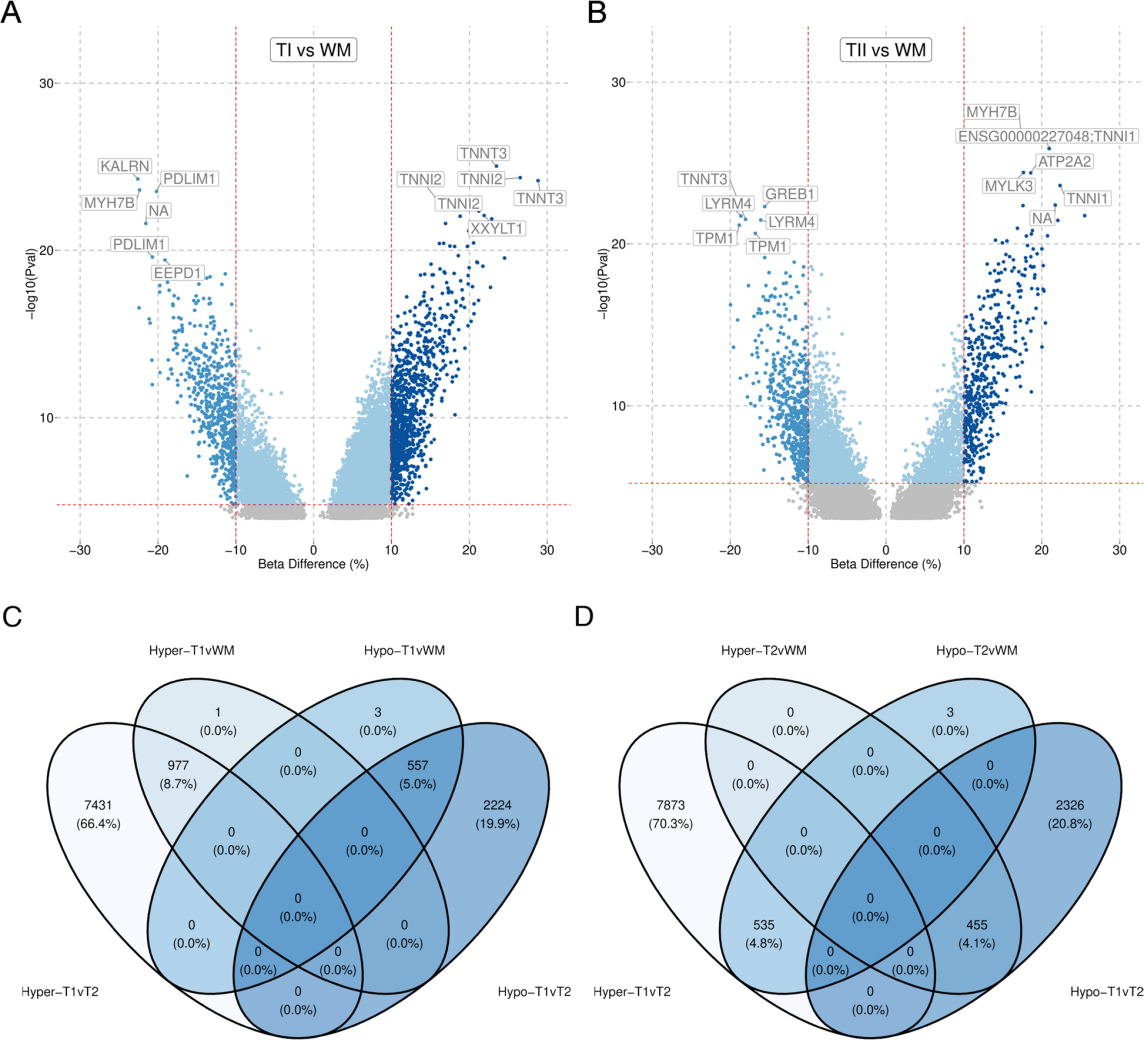
Whole muscle DNAm signature is strongly influenced by TI and TII fibres. **A** Volcano plot of DMPs identified between type I (TI) fibres and whole muscle (WM), with beta difference on the X-axis and -log10 *p*-value on the Y-axis. Royal blue: hypomethylated, dark blue: hypermethylated in TI fibres compared with WM. The horizontal line represents the cutoff of the corresponding adjusted *p*-value $< 0.001$ and the vertical lines represent the cutoff of the beta difference 0.1 and −0.1. **B** Volcano plot of DMPs identified between type II (TII) fibres and WM, with beta difference on the X-axis and -log10 *p*-value on the Y-axis. Royal blue: hypomethylated, dark blue: hypermethylated in TII compared with WM fibres. The horizontal line represents the cutoff of the corresponding adjusted *p*-value $< 0.001$ and the vertical lines represent the cutoff of the beta difference 0.1 and −0.1. **C** Overlaps between DMPs identified in TI vs TII and DMPs identified in TI vs WM. DMPs counts displayed as numbers with overall percentages of DMPs present in each overlap. **D** Overlaps between DMPs identified in TI vs TII and DMPs identified in TII vs WM. DMPs counts displayed as numbers with overall percentages of DMPs present in each overlap

To examine the influence of the fibre types on whole muscle DNAm patterns, we compared the TI fibres with the WM samples and the the TII fibres with the WM samples. A comparison of TI and WM samples at baseline revealed 12,809 DMPs at a BH-FDR $p < 0.001$, of which 1,571 had $>10$% beta difference (hypermethylated = 1000 and hypomethylated = 571) (Fig. 6A). The top three hypomethylated DMPs were *KALRN*, *MYH7B* and *PDLIM1* and the top three hypermethylated DMPs were *TNNT3*, *TNNI2* and *TNNT3* from the TI vs WM comparison (Fig. 6A). A comparison of TII with WM samples revealed 4,897 DMPs at a BH-FDR $p < 0.001$, of which 1007 had $>10$% beta difference (hypermethylated 454 and hypomethylated 553) (Fig. 6B). The top three hypomethylated DMPs were *GREB1*, *LYRM4* and *TNNT3* and the top three hypermethylated DMPs were *MYH7B*, *TNNI1* and *ATP2A2* (Fig. 6B). TI vs TII hypermethylated DMPs overlapped with hypermethylated TI vs WM DMPs and TI vs TII hypomethylated DMPs overlapped with the TI vs WM hypomethylated DMPs (Fig. 6C). In contrast TI vs TII hypermethylated DMPs overlapped with T2vsWM hypomethylated DMPs and TI vs TII hypomethylated DMPs overlapped with the TII vs WM hypomethylated DMPs (Fig. 6D).

These data provide compelling evidence that the whole muscle DNAm signature sits in the middle of TI and TII DNAm signatures, and suggests WM DNAm is strongly influenced by both TI and TII skeletal muscle fibres.

### Estimation of TI and TII fibre proportions using reference-based deconvolution of whole muscle

**Fig. 7:**
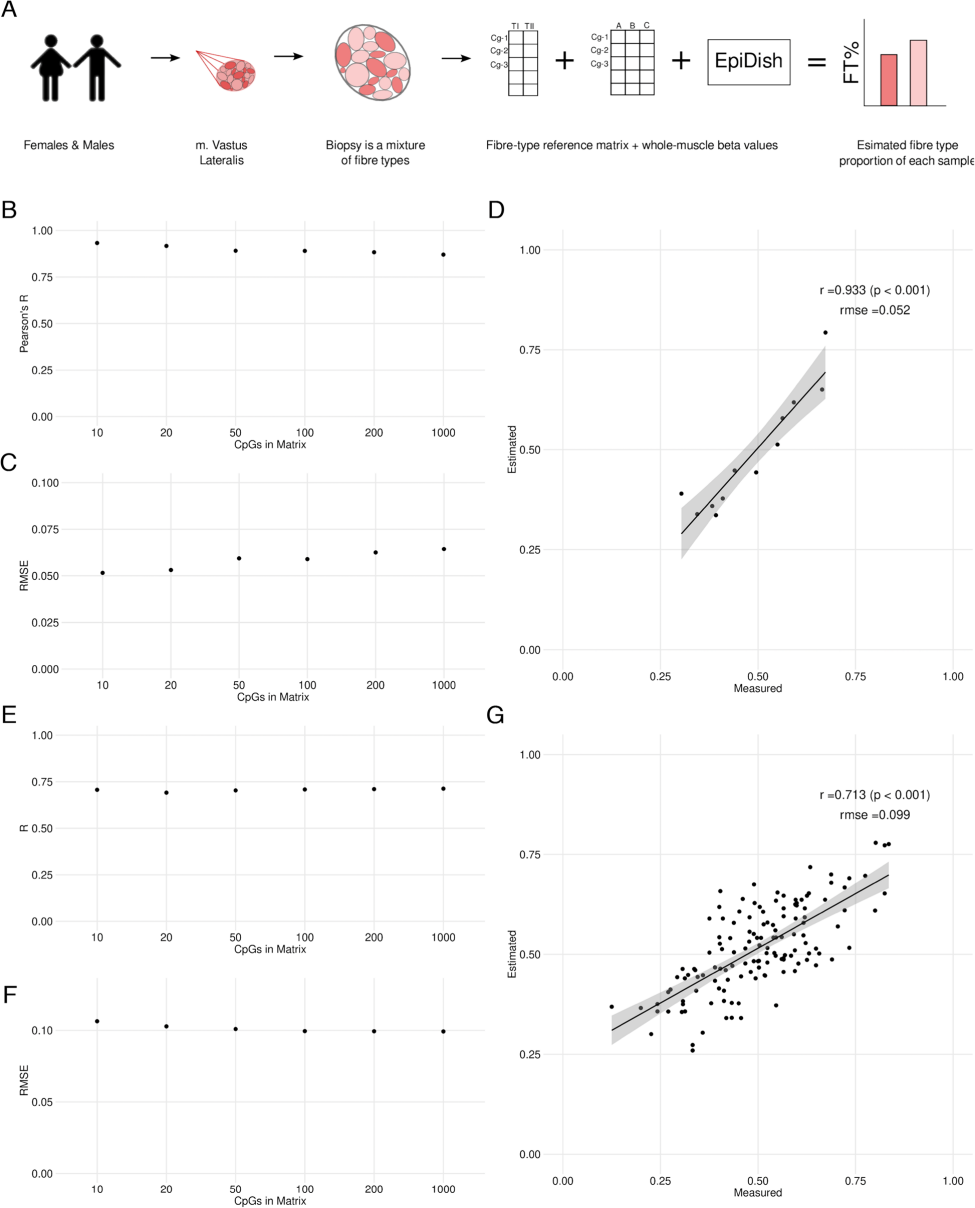
Estimation of TI and TII fibre proportions using reference-based deconvolution of whole muscle. **A** Visual overview of fibre-type deconvolution of whole muscle samples. **B** Pearson’s correlation coefficients of six different fibre-type (FT) reference matrices calculated between measured FT proportions and estimated FT proportions ($n=12$ samples). **C** RMSE values of six different FT reference matrices calculated between measured FT proportions and estimated FT proportions ($n=12$ samples). **D** Scatter plot showing the relationship between measured (FT) proportions and estimated FT proportions using the 10 CpG reference matrix. The figure shows a strong correlation between measured and estimated FT proportions [$r = 0.933$, $p < 0.001$, RMSE = 0.052]. **E** Pearson’s correlation coefficients of six different FT reference matrices calculated between measured FT proportions and estimated FT proportions ($n=171$ samples from the GeneSMART study). **F** RMSE values of six different FT reference matrices calculated between measured FT proportions and estimated FT proportions ($n=174$ samples from the GeneSMART study). **G** Scatter plot showing the relationship between measured FT proportions and estimated FT proportions using the 100 CpG reference matrix. The figure shows a strong correlation between measured and estimated FT proportions using the GeneSMART samples [$r = 0.708$, $p < 0.001$, RMSE = 0.099]

We leveraged our fibre-type DNAm data to generate a reference matrix of TI and TII skeletal muscle fibres that could be used with the EpiDISH [18] package in R to estimate fibre type proportions from WM DNAm data (Fig. 7A). We ranked our identified DMPs with $>10$% beta methylation differences based on a combination of mean beta differences and adjusted *p*-values. We selected the top hypermethylated and hypomethylated DMPs in equal ratios and generated fibre-type DNAm matrices with 10, 20, 50, 100, 200 and 1000 DMPs. We tested each methylation matrix on our WM DNAm samples (as measured with the EPICv2) for which we had TI and TII proportions as determined by single fibre dot blotting from over 200 fibres per sample (Fig. S2A-E). 10 CpGs in the DNAm reference matrix resulted in optimal predictions with the highest Pearson’s correlation (Fig. 7B) and lowest RMSE values (Fig. 7C). The 10 CpG matrix resulted in a very strong correlation, r(10) = 0.933, $p <0.001$, RMSE = 0.052 between the measured proportions of TI and TII fibres with the predicted values (Fig. 7D).

We validated the reference matrices by applying them to 174 GeneSMART WM samples (as measured with the EPICv1) generated in our laboratory [8, 51]. We again tested different numbers of CpGs for deconvolution (10, 20, 50, 100, 200, 1000) (Fig. S2F-J). In contrast to the training samples, 1,000 CpGs in the reference matrix performed best in the validation dataset, showing the highest Pearson’s correlation (Fig. 7E) and lowest RMSE values (Fig. 7F). We observed a strong correlation, r(172) = 0.713, $p <0.001$, RMSE = 0.099 between the measured proportions of TI and TII fibres with the predicted values (Fig. 7G). Using our DNAm profiles of TI and TII muscle fibres we constructed a DNAm reference matrix for the estimation of TI and TII muscle fibre proportions from WM DNAm data. The deconvolution of samples performed well on both the EPICv1 and EPICv2 platforms. Additionally, we have created a web-based tool, MyoTYPE https://myometh.net/myotype, for fibre-type estimation of DNAm data generated with Infinium arrays that enables the rapid typing of muscle samples. For those wishing to apply the fibre-type reference matrix directly in R using EpiDISH, we have provided the reference matrix including the top 1000 DMPs in Additional file 10.

### Exploratory analysis of sex differences in DNAm between TI and TII fibres

**Fig. 8:**
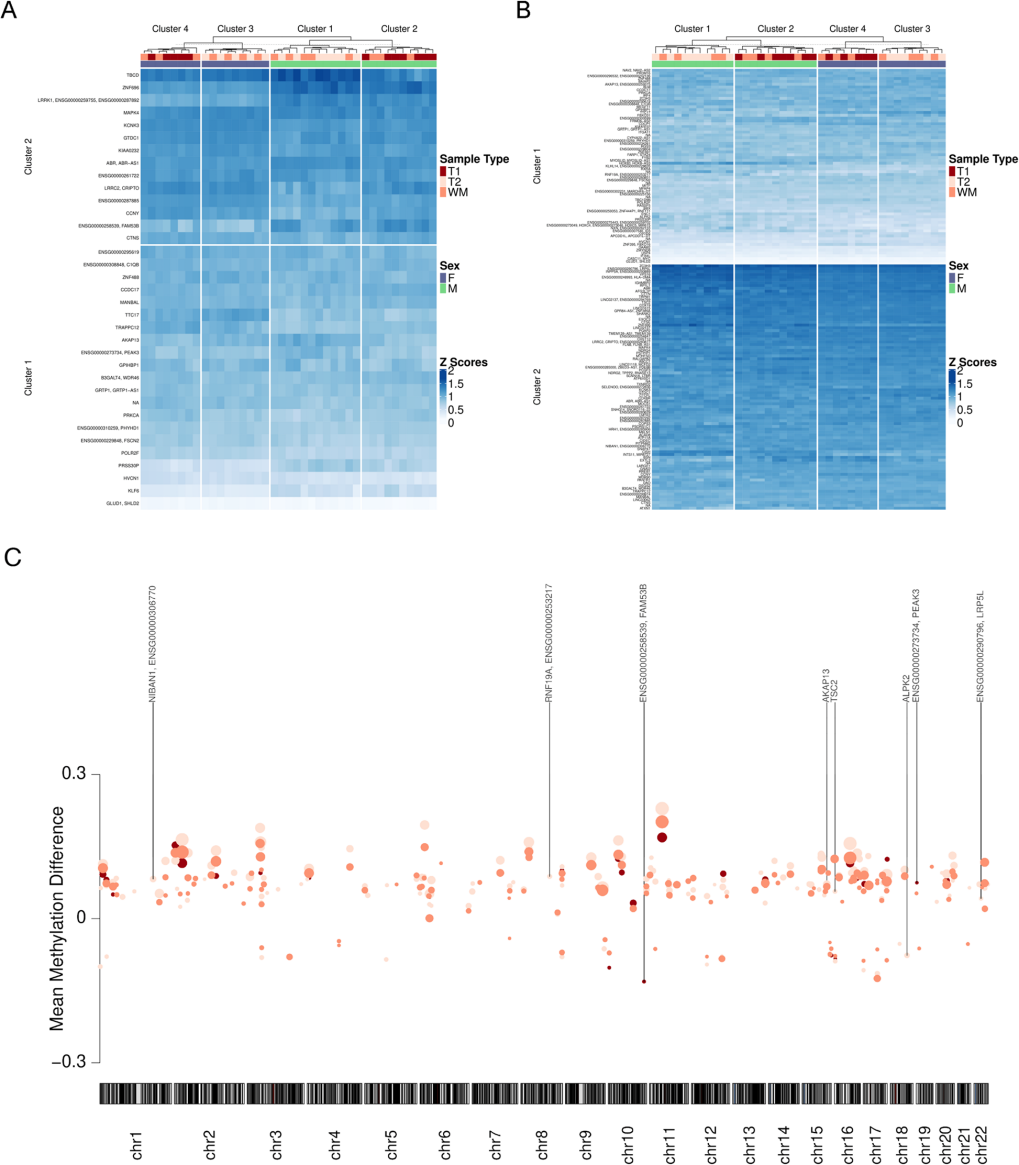
Exploratory analysis of sex differences in DNAm between TI and TII fibres. **A** Heatmap of Z-scores for the 31 DMRs identified in type I (TI) fibres between females and males. Red: TI oxidative fibres, white: type II (TII) glycolytic fibres, orange: whole muscle (WM) samples, purple: females, green: males. **B** Heatmap of Z-scores for the 103 DMRs identified in TI fibres between females and males. Red: TI oxidative fibres, white: TII glycolytic fibres, orange: WM samples, purple: females, green: males. **C** Visual representation of the autosomal location of robust DMRs between females and males. Red: DMRs identified in TI fibres between females and males, orange: DMRs identified in WM between females and males dark beige: DMRs identified in TII fibres between females and males. Mean methylation difference between TI and TII fibres are on the Y-axis with genomic location on the X-axis. Size of the dots represent adjusted *p*-value with larger dots representing a smaller adjusted *p*-value. Gene symbols identify the top four DMRs in each comparison

We conducted an analysis of the sex differences in methylation and protein abundance between TI and TII muscle fibres and WM. A comparison of TI fibres between females and males revealed 757 and 499 (25 hypomethylated and 474 hypermethylated) robustDMPs with a BH-FDR $p < 0.005$ and $< 0.001$ respectively (Fig. S3A and Supplementary Table S6). Further analysis revealed 35 robustDMRs in TI fibres between males and females with 30 hypermethylated and 5 hypomethylated in females compared with males (Fig. 8A).

A comparison of TII fibres between females and males revealed 3,721 DMPs and 2,610 (151 hypomethylated and 2,459 hypermethylated) with a BH-FDR $p < 0.005$ and $< 0.001$ respectively (Fig. S3B and Supplementary Table S7). We identified 156 robustDMRs in TII fibres between males and females with 142 hypermethylated and 14 hypomethylated in females compared with males (Fig. 8B).

A similar analysis of WM samples between females and males revealed 3,455 and 2,244 DMPs (77 hypomethylated 2,167 hypermethylated) with a BH-FDR $p < 0.005$ and $< 0.001$ respectively (Fig. S3C Supplementary Table S8). We identified 144 robustDMRs in WM, with 122 regions hypermethylated and 22 hypomethylated in females compared with males.

Hierarchical k-means clustering of the DMRs in TI and TII fibres separated clearly according to sex (Fig. 8A, B). Overall, we observed a greater hypermethylation in females compared to males in TI fibres, TII fibres and WM samples in line with previously published work [51].

We assessed the overlaps of the robustDMRs from the three comparisons. A total of 26 robustDMRs overlapped all conditions, suggesting differential regulation between the sexes independent of fibre type. We identified 3 robustDMRs specific to the TI FvM comparison, which were annotated to regions in the genes *AKAP13*, *FAM53B* and *PEAK3* (Fig. 8C). Conversely, 60 robustDMRs were identified in the TII FvM comparison, annotated to regions in genes such as *LRP5L*, *TSC2*, *ALPK2*, *NIBAN1* and *RNF19A* (Fig. 8C). We also modelled interaction effects to identify fibre-type-specific sex differences in DNAm. We found 57 and 32 DMPs with a BH-FDR *p* values $< 0.005$ and $< 0.001$ respectively (Fig. S3d and Supplementary Table S9). We identified 3 DMRs that were differentially methylated between fibre types and sexes located in the genes, *CIDEC*, *TDGF1* and *NAV2*. Individual and combined gene set enrichment analysis of the robustDMPs revealed no significant GO or Reactome terms from our list of robustDMPs.

A comparison of female vs male TI fibres at the protein level revealed no DEPs (BH-FDR $p < 0.005$). We identified ALDH2 and ALDH1A1 as DEPs in a female vs male comparison of TII fibres (BH-FDR $p < 0.005$). ALDH2 was up-regulated and ALDH1A1 was down-regulated in TII fibres in females compared with males. ALDH1A1 has been previously identified as differentially regulated between males and females [51]. We identified one DEP annotated to EIF2S1 having an interaction effect between sex and fibre-type with an up-regulation in female TII and a down-regulation in female TI fibres compared with males.

In summary we identified a number of robustDMRs between females and males that were fibre-type independent and a small number of DMPs that differed between the sexes in a fibre-type specific manner. There is a need to include females more frequently when designing experiments investigating skeletal muscle. Our primary outcome from this work was the assessment of DNAm and protein differences between TI and TII skeletal muscle fibres in both males and females, therefore, we were limited to a smaller sample size ($n=7$ males vs $n=5$ females). Sex analysis results can be found in Additional file 11.

## Discussion

We have provided evidence that DNAm patterns are highly fibre-type specific and are associated with protein expression. The application of our method to simultaneously prepare DNA and proteins from the same pure slow-twitch (TI) and fast-twitch (TII) pooled fibres has provided a resource (https://myometh.net) for further investigation. Furthermore, our novel reference matrix enables rapid TI and TII fibre typing of whole muscle DNAm samples.

Previous assessments of fibre-type DNAm in humans were limited to one sample of each fibre type aggregated from male participants and implemented Reduced Representation Bisulfite Sequencing (RRBS) at reduced precision [14]. Here, we measured DNAm from multiple TI ($n=12$) and TII ($n=12$) human muscle fibre samples using the new EPICv2 Infinium array with high precision. We showed that there are considerable differences in DNAm between TI and TII muscle fibres. The influence of fibre-type on the WM methylation signal could be attenuated by the nuclei in the sample coming from cells other than muscle fibres [52, 53]. However our data suggests, that fibre-specific methylation contributes substantially to the whole muscle methylation profile. We found differentially methylated CpGs between muscle fibres in the canonical muscle contraction genes *MYH7*, *TPM3*, *ATP2A2* (TI) and *MYH2*, *TPM1*, *ATP2A1* (TII) which help confer the fibres with their slow and fast contractile phenotypes respectively. These findings agree with those previously reported in humans [14] and in mice [54]. In addition, we have generated a list of robust DMRs (Fig. 3a, b) with at least 4 CpG sites that were differentially methylated between TI and TII muscle fibres. We identified *MAD1L1*, which has recently been reported as a DMR in whole skeletal muscle between groups exercising in either energy deficit or energy surplus [55], as having multiple hypermethylated regions in TI fibres vs TII fibres. It may be that this DMR in whole muscle is driven by fibre-type proportions of samples rather than by exercise. We envision that this data repository may provide useful insights for researchers who would like to explore the fibre-type DNAm patterns of specific genes.

The coordination of DNA, RNA and protein is of major interest [56] and as such the integration of multiple OMICs in skeletal muscle is often performed [7–10, 51, 57]. However, the study of the diverse biological layers in WM or muscle fibre types are conducted either in isolation [15, 17], or from different muscle samples often collected from different cohorts, leading to less accurate comparisons [8, 9, 14, 51]. We measured protein abundance in the same pooled fibre samples and showed that MYH7, MYH2, TNNI1, TNNT1, TNNT3, ATP2A2 and ATP2A1 were all differentially regulated between fibre types in the proteome, which aligned with the results from the methylome. These results are in agreement with those presented in previous methylome and proteome studies of human skeletal muscle fibre types [14, 15, 17]. Furthermore, we identified key metabolism Reactome pathways such as glycolysis, metabolism of carbohydrates, glycogen metabolism and glycogen breakdown in the overlapping hypermethylated DMPs and down regulated DEPs from TI compared with TII fibres. It has previously been reported that TII muscle fibres rely more on glycolytic energy pathways than TI fibres (reviewed in [1, 58, 59]). We have also shown a strong association between DNAm and protein expression in human muscle and suggest that hypomethylation in glycolytic pathway genes in TII muscle fibres prepares them for specific energy demands compared with TI fibres.

Understanding cell-type populations and their proportions in samples is crucial when designing DNAm studies [60]. To overcome cell-type confounding, referenced-based deconvolution of DNAm data has been developed for other tissues, allowing statistical correction of cell-type proportions [61]. Our data suggests whole-muscle (WM) DNAm signatures are strongly influenced by TI and TII skeletal muscle fibre types. The proportions of fibre-types in human skeletal muscle can vary considerably between individuals and even from different biopsies taken from the same individual [62], and there is a significant influence of fibre-type proportions on DNAm analyses in skeletal muscle [11]. The influence of fibre-type on skeletal muscle DNAm has been accounted for by including fibre-type as a covariate in modelling [11]. Our study underscores the significant influence of fibre-type on DNAm signatures in skeletal muscle, highlighting the acute need to adjust for fibre-type proportions in WM DNAm studies. To address this, we have established a novel reference matrix for the deconvolution of whole skeletal muscle DNAm data aimed at approaching the challenge of understanding the influence of cell-type proportions of whole muscle tissue samples [63, 64]. Our methylation matrix can be applied within the EpiDISH [18] framework to estimate TI and TII proportions of skeletal muscle samples. A benefit of this reference-matrix is that it could account for the variation in fibre-type proportions between muscle biopsies by providing sample specific fibre-type proportion estimates for model fitting.

Most of the previous studies on fibre-type specific regulation have been limited by including male participants only, leading to an underrepresentation of females. Considering there is evidence that there are sex differences in muscle function [65] and muscle fibre types [66], there is a need to include females in study designs. This limitation has been overcome by recruitment of both males and females, and the generation of fibre-type specific DNAm data that is more representative of the population. In agreement with previous work from our laboratory [51], there was hypermethylation of whole muscle (WM) in females and, interestingly, hypermethylation in TI and TII fibres in females compared with males. In addition, we found a larger number of robustDMPs in TII fibres between females and males than in TI fibres between females and males. A number of DMRs that we identified as differentially methylated in female TI fibres vs male TI fibres, such as PEAK3 [67], AKAP13 [68] (hypermethylated) and FAM53B [69] (hypomethylated), are mediators of cell signalling pathways that may play a role in TI fibre size differences between males and females. We observed several sex differences in DNAm independent of fibre type, however, a subset of DNAm sites differed between the sexes in a fibre-type specific manner. This suggests that although fibre-type plays a smaller than expected role in the sex differences in DNAm in skeletal muscle, a subset of fibre-type sex-specific DNAm differences may remain unidentified in WM DNAm studies (Fig. S3).

### Limitations

We have identified a number of limitations in this work. Firstly, skeletal muscle is comprised of cells other than muscle fibres with approximately 50-70% of muscle nuclei coming from myonuclei [52, 53]. Furthermore, single fibre imaging has shown that satellite cells and endothelial cells have the potential to line single skeletal muscle fibres [70] therefore we cannot exclude the possibility that these cells may be contaminating our pooled fibres. Future work could adapt our method to prepare single myonuclei from pooled muscle fibre types. Secondly, we analysed slow TI and fast TII fibre populations but were unable to analyse pure TIIa, and TIIx fibres nor hybrid fibre populations. Thirdly, recent work indicates that there is considerable heterogeneity within muscle fibre types [16, 71]. Due to the pooling strategy, we were unable to resolve differences between fibres within a TI or TII population. And finally, we did not perform transcriptomic analyses due to lack of extra muscle samples. In the future, it would be of interest to integrate fibre-specific transcriptomic, DNAm, proteomics and metabolomics data to offer a more complete understanding of skeletal muscle biology. This work provides a platform for the preparation of DNA, RNA and protein from the same sample.

## Conclusion

We investigated DNAm and protein expression simultaneously from pooled TI and TII human skeletal muscle fibre samples and whole-muscle in males and females. We report considerable DNAm differences between TI and TII fibres and show evidence that DNAm patterns in whole muscle are an average of TI and TII fibre signatures, suggesting fibre-type proportion of samples can impact results. There were relationships between DNAm and protein abundance in key contractile and metabolic pathways. Furthermore, we have constructed a novel DNAm reference matrix that enables TI and TII fibre-type proportion estimations of whole muscle DNAm samples. We provide the methylome and proteome profiles presented here and corresponding deconvolution tool MyoTYPE as a resource for the research community. This can be accessed and freely used via a web interface we developed namely MyoMETH https://myometh.net.

## Supplementary information

Additional file 1. Supplementary Figures S1-S3 and Supplementary Tables S1-S9.

Additional file 2. Unique peptides used for myosin isoform analysis.

Additional file 3. Myosin isoform analysis results.

Additional file 4. Proteome differential analysis results.

Additional file 5. Combined Ion file.

Additional file 6. Modified peptides file.

Additional file 7. Combined peptide file.

Additional file 8. Combined protein file.

Additional file 9. Methylome differential analysis results.

Additional file 10. Fibre-Type DNAm reference matrix.

Additional file 11. Sex differences analysis results.

## Data Availability

Data availability: The mass spectrometry proteomics data have been deposited to the ProteomeXchange http://proteomecentral.proteomexchange.org Consortium via the PRIDE [72] partner repository with the dataset identifier PXD066393. The methylation data generated in this publication has been deposited in NCBI’s Gene Expression Omnibus [73, 74] and are accessible through GEO Series accession number GSE304045 https://www.ncbi.nlm.nih.gov/geo/query/acc.cgi?acc=GSE304045. Code availability: Custom R code used in this analysis are publicly available at https://github.com/AndrewStuartPalmer/palmer-2025-integrated-fibre-specific-methylome-proteome. A computational docker container of the R environment is also available https://hub.docker.com/r/andrewspalmer/r-studio-computational-env-palmer-2025.

## References

[1] Schiaffino S, Reggiani C. Fiber types in mammalian skeletal muscles. Physiol Rev. 2011;91(4):1447–531. 10.1152/physrev.00031.2010. 22013216 10.1152/physrev.00031.2010

[2] Moore LD, Le T, Fan G. DNA methylation and its basic function. Neuropsychopharmacology. 2012;38(1):23–38. 10.1038/npp.2012.112. 22781841 10.1038/npp.2012.112 PMC3521964

[3] Smith ZD, Meissner A. DNA methylation: roles in mammalian development. Nat Rev Genet. 2013;14(3):204–20. 10.1038/nrg3354. 23400093 10.1038/nrg3354

[4] Zykovich A, Hubbard A, Flynn JM, Tarnopolsky M, Fraga MF, Kerksick C, et al. Genome-wide DNA methylation changes with age in disease-free human skeletal muscle. Aging Cell. 2013;13(2):360–6. 10.1111/acel.12180. 24304487 10.1111/acel.12180 PMC3954952

[5] Lindholm ME, Marabita F, Gomez-Cabrero D, Rundqvist H, Ekström TJ, Tegnér J, et al. An integrative analysis reveals coordinated reprogramming of the epigenome and the transcriptome in human skeletal muscle after training. Epigenetics. 2014;9(12):1557–69. 10.4161/15592294.2014.982445. 25484259 10.4161/15592294.2014.982445 PMC4622000

[6] Seaborne RA, Strauss J, Cocks M, Shepherd S, O’Brien TD, van Someren KA, et al. Human skeletal muscle possesses an epigenetic memory of hypertrophy. Sci Rep. 2018;8(1):1898. 10.1038/s41598-018-20287-3. 29382913 10.1038/s41598-018-20287-3 PMC5789890

[7] Voisin S, Jacques M, Landen S, Harvey NR, Haupt LM, Griffiths LR, et al. Meta-analysis of genome-wide DNA methylation and integrative omics of age in human skeletal muscle. J Cachexia Sarcopenia Muscle. 2021;12(4):1064–78. 10.1002/jcsm.12741. 34196129 10.1002/jcsm.12741 PMC8350206

[8] Jacques M, Landen S, Romero JA, Hiam D, Schittenhelm RB, Hanchapola I, et al. Methylome and proteome integration in human skeletal muscle uncover group and individual responses to high-intensity interval training. FASEB J. 2023. 10.1096/fj.202300840rr. 37698381 10.1096/fj.202300840RR

[9] Voisin S, Seale K, Jacques M, Landen S, Harvey NR, Haupt LM, et al. Exercise is associated with younger methylome and transcriptome profiles in human skeletal muscle. Aging Cell. 2023. 10.1111/acel.13859. 37128843 10.1111/acel.13859 PMC10776126

[10] Landen S, Jacques M, Hiam D, Alvarez-Romero J, Schittenhelm RB, Shah AD, et al. Sex differences in muscle protein expression and DNA methylation in response to exercise training. Biol Sex Differ. 2023;14(1):56. 10.1186/s13293-023-00539-2. 37670389 10.1186/s13293-023-00539-2 PMC10478435

[11] Taylor DL, Jackson AU, Narisu N, Hemani G, Erdos MR, Chines PS, et al. Integrative analysis of gene expression, DNA methylation, physiological traits, and genetic variation in human skeletal muscle. Proc Natl Acad Sci U S A. 2019;116(22):10883–8. 10.1073/pnas.1814263116. 31076557 10.1073/pnas.1814263116 PMC6561151

[12] Raue U, Trappe TA, Estrem ST, Qian HR, Helvering LM, Smith RC, et al. Transcriptome signature of resistance exercise adaptations: mixed muscle and fiber type specific profiles in young and old adults. J Appl Physiol. 2012;112(10):1625–36. 10.1152/japplphysiol.00435.2011. 22302958 10.1152/japplphysiol.00435.2011 PMC3365403

[13] Murgia M, Nagaraj N, Deshmukh AS, Zeiler M, Cancellara P, Moretti I, et al. Single Muscle Fiber Proteomics Reveals Unexpected Mitochondrial Specialization. EMBO Rep. 2015;16(3):387–395. 10.15252/embr.201439757. 10.15252/embr.201439757 PMC4364878 25643707

[14] Begue G, Raue U, Jemiolo B, Trappe S. DNA methylation assessment from human slow- and fast-twitch skeletal muscle fibers. J Appl Physiol. 2017;122(4):952–67. 10.1152/japplphysiol.00867.2016. 28057818 10.1152/japplphysiol.00867.2016 PMC5407195

[15] Deshmukh AS, Steenberg DE, Hostrup M, Birk JB, Larsen JK, Santos A, et al. Deep muscle-proteomic analysis of freeze-dried human muscle biopsies reveals fiber type-specific adaptations to exercise training. Nat Commun. 2021;12(1):304. 10.1038/s41467-020-20556-8. 33436631 10.1038/s41467-020-20556-8 PMC7803955

[16] Murgia M, Nogara L, Baraldo M, Reggiani C, Mann M, Schiaffino S. Protein profile of fiber types in human skeletal muscle: a single-fiber proteomics study. Skelet Muscle. 2021;11(1):24. 10.1186/s13395-021-00279-0. 34727990 10.1186/s13395-021-00279-0 PMC8561870

[17] Reisman EG, Botella J, Huang C, Schittenhelm RB, Stroud DA, Granata C, et al. Fibre-specific mitochondrial protein abundance is linked to resting and post-training mitochondrial content in the muscle of men. Nat Commun. 2024;15(1):7677. 10.1038/s41467-024-50632-2. 39227581 10.1038/s41467-024-50632-2 PMC11371815

[18] Zheng SC, Breeze CE, Beck S, Teschendorff AE. Identification of differentially methylated cell types in epigenome-wide association studies. Nat Methods. 2018;15(12):1059–66. 10.1038/s41592-018-0213-x. 30504870 10.1038/s41592-018-0213-x PMC6277016

[19] Yan X, Eynon N, Papadimitriou ID, Kuang J, Munson F, Tirosh O, et al. The gene smart study: method, study design, and preliminary findings. BMC Genomics. 2017;18(S8):821. 10.1186/s12864-017-4186-4. 29143594 10.1186/s12864-017-4186-4 PMC5688409

[20] Mifflin M, Jeor SS, Hill L, Scott B, Daugherty S, Koh Y. A new predictive equation for resting energy expenditure in healthy individuals. Am J Clin Nutr. 1990;51(2):241–7. 10.1093/ajcn/51.2.241. 2305711 10.1093/ajcn/51.2.241

[21] National Health and Medical Research Council. Australian Dietary Guidelines: Providing the Scientific Evidence for Healthier Australian Diets. https://www.nhmrc.gov.au/_files_nhmrc/file/publications/n55_australian_dietary_guidelines1.pdf. Accessed 07 Oct 2023.

[22] Bergström J. Muscle electrolytes in man: determined by neutron activation analysis on needle biopsy specimens. Scand J Clin Lab Investig (Engl). 1962;14:511–3.

[23] Christiansen D, MacInnis MJ, Zacharewicz E, Xu H, Frankish BP, Murphy RM. A fast, reliable and sample-sparing method to identify fibre types of single muscle fibres. Sci Rep. 2019;9(1):6473. 10.1038/s41598-019-42168-z. 31019216 10.1038/s41598-019-42168-z PMC6482153

[24] Gautam A. 3. In: Phenol-Chloroform DNA Isolation Method. DNA and RNA Isolation Techniques for Non-Experts. Springer International Publishing; 2022. p. 33–39. 10.1007/978-3-030-94230-4_3.

[25] Buhule OD, Minster RL, Hawley NL, Medvedovic M, Sun G, Viali S, et al. Stratified Randomization Controls Better for Batch Effects in 450k Methylation Analysis: a Cautionary Tale. Front Genet. 2014;5(nil):nil.10.3389/fgene.2014.00354. 10.3389/fgene.2014.00354 PMC4195366 25352862

[26] Mansell G, Gorrie-Stone TJ, Bao Y, Kumari M, Schalkwyk LS, Mill J, et al. Guidance for DNA methylation studies: statistical insights from the Illumina EPIC array. BMC Genomics. 2019;20(1):366. 10.1186/s12864-019-5761-7. 31088362 10.1186/s12864-019-5761-7 PMC6518823

[27] R Core Team.: R: A Language and Environment for Statistical Computing. Vienna. http://www.R-project.org/. Accessed 31 Oct 2022.

[28] Aryee MJ, Jaffe AE, Corrada-Bravo H, Ladd-Acosta C, Feinberg AP, Hansen KD, et al. Minfi: a flexible and comprehensive bioconductor package for the analysis of Infinium DNA methylation microarrays. Bioinformatics. 2014;30(10):1363–9. 10.1093/bioinformatics/btu049. 24478339 10.1093/bioinformatics/btu049 PMC4016708

[29] Pidsley R, Wong CCY, Volta M, Lunnon K, Mill J, Schalkwyk LC. A data-driven approach to preprocessing Illumina 450k methylation array data. BMC Genomics. 2013;14(1):293. 10.1186/1471-2164-14-293. 23631413 10.1186/1471-2164-14-293 PMC3769145

[30] Pidsley R, Zotenko E, Peters TJ, Lawrence MG, Risbridger GP, Molloy P, et al. Critical evaluation of the Illumina MethylationEpic BeadChip microarray for whole-genome DNA methylation profiling. Genome Biol. 2016;17(1):208. 10.1186/s13059-016-1066-1. 27717381 10.1186/s13059-016-1066-1 PMC5055731

[31] an Chen Y, Lemire M, Choufani S, Butcher DT, Grafodatskaya D, Zanke BW, et al. Discovery of Cross-Reactive Probes and Polymorphic Cpgs in the Illumina Infinium Humanmethylation450 Microarray. Epigenetics. 2013;8(2):203–209. 10.4161/epi.23470. 10.4161/epi.23470 PMC3592906 23314698

[32] Peters TJ, Meyer B, Ryan L, Achinger-Kawecka J, Song J, Campbell EM, et al. Characterisation and reproducibility of the HumanMethylationEPIC V2.0 BeadChip for DNA methylation profiling. BMC Genomics. 2024;25(1):251. 10.1186/s12864-024-10027-5. 38448820 10.1186/s12864-024-10027-5 PMC10916044

[33] Peters TJ, Buckley MJ, Statham AL, Pidsley R, Samaras K, Lord RV, et al. De novo identification of differentially methylated regions in the human genome. Epigenetics Chromatin. 2015;8(1):6. 10.1186/1756-8935-8-6. 25972926 10.1186/1756-8935-8-6 PMC4429355

[34] Xu Z, Niu L, Taylor JA. The enmix DNA methylation analysis pipeline for Illumina Beadchip and comparisons with seven other preprocessing pipelines. Clin Epigenetics. 2021;13(1):216. 10.1186/s13148-021-01207-1. 34886879 10.1186/s13148-021-01207-1 PMC8662917

[35] Smyth GK. Linear models and empirical Bayes methods for assessing differential expression in microarray experiments. Stat Appl Genet Mol Biol. 2004;3(1):1–25. 10.2202/1544-6115.1027. 10.2202/1544-6115.1027 16646809

[36] Ritchie ME, Phipson B, Wu D, Hu Y, Law CW, Shi W, et al. Limma powers differential expression analyses for Rna-sequencing and microarray studies. Nucleic Acids Res. 2015;43(7):e47–e47. 10.1093/nar/gkv007. 25605792 10.1093/nar/gkv007 PMC4402510

[37] Benjamini Y, Hochberg Y. Controlling the false discovery rate: a practical and powerful approach to multiple testing. J R Stat Soc Ser B Methodol. 1995;57(1):289–300. 10.1111/j.2517-6161.1995.tb02031.x.

[38] Phipson B, Maksimovic J, Oshlack A. Missmethyl: an R package for analyzing data from Illumina’s Humanmethylation450 platform. Bioinformatics. 2015;32(2):286–8. 10.1093/bioinformatics/btv560. 26424855 10.1093/bioinformatics/btv560

[39] Wiśniewski JR, Zougman A, Nagaraj N, Mann M. Universal sample preparation method for proteome analysis. Nat Methods. 2009;6(5):359–62. 10.1038/nmeth.1322. 19377485 10.1038/nmeth.1322

[40] Kulak NA, Pichler G, Paron I, Nagaraj N, Mann M. Minimal, encapsulated proteomic-sample processing applied to copy-number estimation in eukaryotic cells. Nat Methods. 2014;11(3):319–24. 10.1038/nmeth.2834. 24487582 10.1038/nmeth.2834

[41] Zolg DP, Wilhelm M, Schnatbaum K, Zerweck J, Knaute T, Delanghe B, et al. Building proteometools based on a complete synthetic human proteome. Nat Methods. 2017;14(3):259–62. 10.1038/nmeth.4153. 28135259 10.1038/nmeth.4153 PMC5868332

[42] Zauber H, Kirchner M, Selbach M. Picky: a simple online Prm and Srm method designer for targeted proteomics. Nat Methods. 2018;15(3):156–7. 10.1038/nmeth.4607. 29489744 10.1038/nmeth.4607

[43] Kong AT, Leprevost FV, Avtonomov DM, Mellacheruvu D, Nesvizhskii AI. Msfragger: ultrafast and comprehensive peptide identification in mass spectrometry-based proteomics. Nat Methods. 2017;14(5):513–20. 10.1038/nmeth.4256. 28394336 10.1038/nmeth.4256 PMC5409104

[44] Yu F, Haynes SE, Nesvizhskii AI. Ionquant enables accurate and sensitive label-free quantification with FDR-controlled match-between-runs. Mol Cell Proteomics. 2021;20:100077. 10.1016/j.mcpro.2021.100077. 33813065 10.1016/j.mcpro.2021.100077 PMC8131922

[45] Fraterman S, Zeiger U, Khurana TS, Wilm M, Rubinstein NA. Quantitative proteomics profiling of sarcomere associated proteins in limb and extraocular muscle allotypes. Mol Cell Proteomics. 2007;6(4):728–37. 17229715 10.1074/mcp.M600345-MCP200

[46] Drexler HC, Ruhs A, Konzer A, Mendler L, Bruckskotten M, Looso M, et al. On marathons and Sprints: an integrated quantitative proteomics and transcriptomics analysis of differences between slow and fast muscle fibers. Mol Cell Proteomics. 2012;11(6):M111-010801. 10.1074/mcp.M111.010801 PMC3433927 22210690

[47] Yu G, Wang LG, Han Y, He QY. Clusterprofiler: an R package for comparing biological themes among gene clusters. OMICS. 2012;16(5):284–7. 10.1089/omi.2011.0118. 22455463 10.1089/omi.2011.0118 PMC3339379

[48] Wu T, Hu E, Xu S, Chen M, Guo P, Dai Z, et al. Clusterprofiler 4.0: a universal enrichment tool for interpreting omics data. Innov (Camb). 2021;2(3):100141. 10.1016/j.xinn.2021.100141. 10.1016/j.xinn.2021.100141 PMC8454663 34557778

[49] Momenzadeh A, Jiang Y, Kreimer S, Teigen LE, Zepeda CS, Haghani A, et al. A complete workflow for high throughput human single skeletal muscle fiber proteomics. J Am Soc Mass Spectrom. 2023;34(9):1858–67. 37463334 10.1021/jasms.3c00072 PMC11135628

[50] Jones PA. Functions of DNA methylation: islands, start sites, gene bodies and beyond. Nat Rev Genet. 2012;13(7):484–92. 10.1038/nrg3230. 22641018 10.1038/nrg3230

[51] Landen S, Jacques M, Hiam D, Alvarez-Romero J, Harvey NR, Haupt LM, et al. Skeletal muscle methylome and transcriptome integration reveals profound sex differences related to muscle function and substrate metabolism. Clin Epigenetics. 2021;13(1):202. 10.1186/s13148-021-01188-1. 34732242 10.1186/s13148-021-01188-1 PMC8567658

[52] Schmalbruch H, Hellhammer U. The number of nuclei in adult rat muscles with special reference to satellite cells. Anat Rec. 1977;189(2):169–75. 10.1002/ar.1091890204. 911042 10.1002/ar.1091890204

[53] Santos MD, Backer S, Saintpierre B, Izac B, Andrieu M, Letourneur F, et al. Single-nucleus Rna-seq and fish identify coordinated transcriptional activity in mammalian myofibers. Nat Commun. 2020;11(1):5102. 10.1038/s41467-020-18789-8. 33037211 10.1038/s41467-020-18789-8 PMC7547110

[54] Oe M, Ojima K, Muroya S. Difference in potential DNA methylation impact on gene expression between fast- and slow-type myofibers. Physiol Genomics. 2021;53(2):69–83. 10.1152/physiolgenomics.00099.2020. 33459151 10.1152/physiolgenomics.00099.2020

[55] Gorski PP, Turner DC, Iraki J, Morton JP, Sharples AP, Areta JL. Human skeletal muscle methylome after low-carbohydrate energy-balanced exercise. Am J Physiol Endocrinol Metab. 2023;324(5):E437–48. 10.1152/ajpendo.00029.2023. 37018654 10.1152/ajpendo.00029.2023

[56] Manzoni C, Kia DA, Vandrovcova J, Hardy J, Wood NW, Lewis PA, et al. Genome, Transcriptome and Proteome: the rise of omics data and their integration in biomedical sciences. Brief Bioinform. 2016;19(2):286–302. 10.1093/bib/bbw114. 10.1093/bib/bbw114 PMC6018996 27881428

[57] Rowlands DS, Page RA, Sukala WR, Giri M, Ghimbovschi SD, Hayat I, et al. Multi-omic integrated networks connect DNA methylation and miRNA with skeletal muscle plasticity to chronic exercise in type 2 diabetic obesity. Physiol Genomics. 2014;46(20):747–65. 10.1152/physiolgenomics.00024.2014. 25138607 10.1152/physiolgenomics.00024.2014 PMC4200377

[58] Pette D. Metabolic heterogeneity of muscle fibres. J Exp Biol. 1985;115(1):179–89. 10.1242/jeb.115.1.179. 4031763 10.1242/jeb.115.1.179

[59] Smith JAB, Murach KA, Dyar KA, Zierath JR. Exercise metabolism and adaptation in skeletal muscle. Nat Rev Mol Cell Biol. 2023;24(9):607–32. 10.1038/s41580-023-00606-x. 37225892 10.1038/s41580-023-00606-x PMC10527431

[60] Lappalainen T, Greally JM. Associating cellular epigenetic models with human phenotypes. Nat Rev Genet. 2017;18(7):441–51. 10.1038/nrg.2017.32. 28555657 10.1038/nrg.2017.32

[61] Teschendorff AE, Breeze CE, Zheng SC, Beck S. A comparison of reference-based algorithms for correcting cell-type heterogeneity in epigenome-wide association studies. BMC Bioinformatics. 2017;18(1):105. 10.1186/s12859-017-1511-5. 28193155 10.1186/s12859-017-1511-5 PMC5307731

[62] Sahl RE, Morville T, Kraunsøe R, Dela F, Helge JW, Larsen S. Variation in Mitochondrial Respiratory Capacity and Myosin Heavy Chain Composition in Repeated Muscle Biopsies. Anal Biochem. 2018;556(nil):119–124. 10.1016/j.ab.2018.06.029. 10.1016/j.ab.2018.06.029 29966588

[63] Jaffe AE, Irizarry RA. Accounting for cellular heterogeneity is critical in epigenome-wide association studies. Genome Biol. 2014;15(2):R31. 10.1186/gb-2014-15-2-r31. 24495553 10.1186/gb-2014-15-2-r31 PMC4053810

[64] Teschendorff AE, Zheng SC. Cell-type deconvolution in epigenome-wide association studies: a review and recommendations. Epigenomics. 2017;9(5):757–68. 10.2217/epi-2016-0153. 28517979 10.2217/epi-2016-0153

[65] Miller AEJ, MacDougall JD, Tarnopolsky MA, Sale DG. Gender differences in strength and muscle fiber characteristics. Eur J Appl Physiol Occup Physiol. 1993;66(3):254–62. 10.1007/bf00235103. 8477683 10.1007/BF00235103

[66] Nuzzo JL. Sex differences in skeletal muscle fiber types: a meta-analysis. Clin Anat. 2023;37(1):81–91. 10.1002/ca.24091. 37424380 10.1002/ca.24091

[67] Roy MJ, Surudoi MG, Kropp A, Hou J, Dai W, Hardy JM, et al. Structural mapping of peak pseudokinase interactions identifies 14-3-3 as a molecular switch for Peak3 signaling. Nat Commun. 2023;14(1):3542. 10.1038/s41467-023-38869-9. 37336884 10.1038/s41467-023-38869-9 PMC10279719

[68] Zhang S, Wang H, Melick CH, Jeong MH, Curukovic A, Tiwary S, et al. Akap13 couples GPcr signaling to mTORc1 inhibition. PLoS Genet. 2021;17(10):e1009832. 10.1371/journal.pgen.1009832. 34673774 10.1371/journal.pgen.1009832 PMC8570464

[69] Kizil C, Küchler B, Yan JJ, Özhan G, Moro E, Argenton F, et al. Simplet/fam53b Is Required for Wnt Signal Transduction By Regulating -catenin Nuclear Localization. Development. 2014;141(18):3529–39. 10.1242/dev.108415. 10.1242/dev.108415 25183871

[70] Gnocchi VF, White RB, Ono Y, Ellis JA, Zammit PS. Further characterisation of the molecular signature of quiescent and activated mouse muscle satellite cells. PLoS ONE. 2009;4(4):e5205. 10.1371/journal.pone.0005205. 19370151 10.1371/journal.pone.0005205 PMC2666265

[71] Moreno-Justicia R, Stede TV, Stocks B, Laitila J, Seaborne RA, Loock AV, et al. Human skeletal muscle fiber heterogeneity beyond myosin heavy chains. Nat Commun. 2025;16(1):1764. 10.1038/s41467-025-56896-6. 39971958 10.1038/s41467-025-56896-6 PMC11839989

[72] Perez-Riverol Y, Bandla C, Kundu DJ, Kamatchinathan S, Bai J, Hewapathirana S, et al. The PRIDE database at 20 years: 2025 update. Nucleic Acids Res. 2025;53(D1):D543–53. 39494541 10.1093/nar/gkae1011 PMC11701690

[73] Edgar R, Domrachev M, Lash AE. Gene Expression Omnibus: NCBI gene expression and hybridization array data repository. Nucleic Acids Res. 2002;30(1):207–10. 11752295 10.1093/nar/30.1.207 PMC99122

[74] Barrett T, Wilhite SE, Ledoux P, Evangelista C, Kim IF, Tomashevsky M, et al. Ncbi geo: archive for functional genomics data sets—update. Nucleic Acids Res. 2012;41(11):D991–5. 10.1093/nar/gks1193. 23193258 10.1093/nar/gks1193 PMC3531084

