# Supplementary material for: Integrated fibre-specific methylome and proteome profiling of human skeletal muscle across males and females with fibre-type deconvolution
Source: Skelet Muscle. 2025 Oct 10;15:28. doi: 10.1186/s13395-025-00396-0 (PMC12512927; doi:10.1186/s13395-025-00396-0)
Supplement: Supplementary file 1 — Additional file 1. Supplementary Figures S1-S3 and Supplementary Tables S1-S9. [file 13395_2025_396_MOESM1_ESM.pdf]

Additional File 1.

Integrated fibre-type-specific methylome and proteome profiling of human skeletal muscle across males and females with fibre-type deconvolution.

Andrew S Palmer *et al.*

This PDF file includes:

Figs. S1 to S3
Tables S1-S9

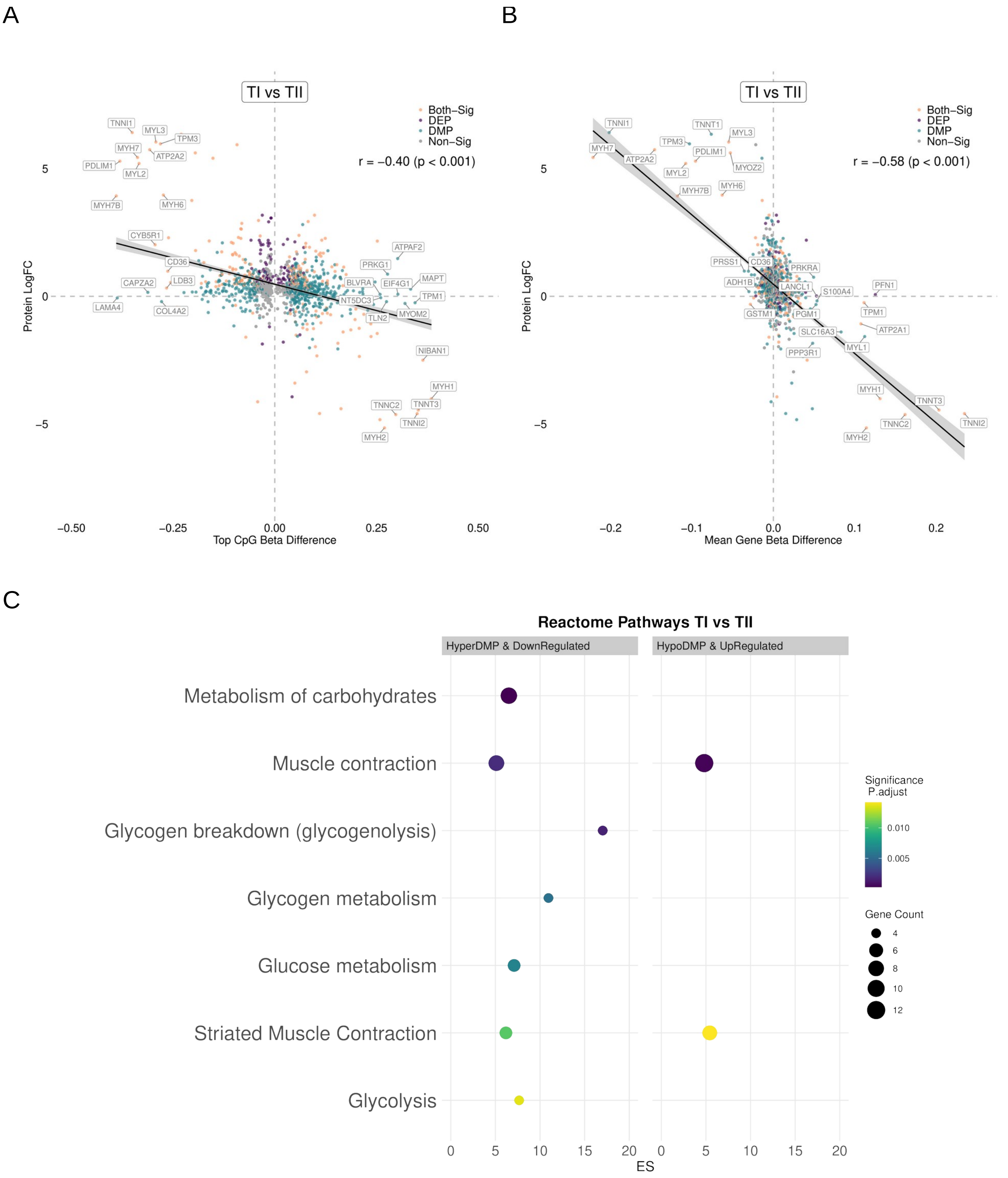

Fig. S1.

Relationship between DMPs and Proteins in TI and TII fibres. **(A)** Pearson's correlation between the beta difference for the top DMP and matched protein logFC [r(1139) = -0.40, p = <0.001].

Purple: only significant DEP, green: only significant DMP, orange: both DMP and DEP significant, grey: both non-significant. The Y-axis is the beta difference between type I (TI) and type II (TII) fibres and the X-axis is the logFC difference of proteins between TI and TII fibres. **(B)** Pearson's correlation between the mean beta difference for all DMPs in each protein and protein logFC in each identified gene with a measured protein [$r(1139) = -0.58$, $p = < 0.001$]. Purple: only significant DEP, green: only significant DMP, orange: both DMP and DEP significant, grey: both non-significant. The Y-axis is the beta difference between TI and TII fibres and the X-axis is the logFC difference of proteins between TI and TII fibres. **(C)** Reactome enrichment analysis of either the HyperDMP and DownRegulated protein pairs or HypoDMP and UpRegulated protein pairs identified between TI and TII fibres. The circle size represents gene counts, and the colour represents the adjusted p-value and the enrichment score on the X-axis.

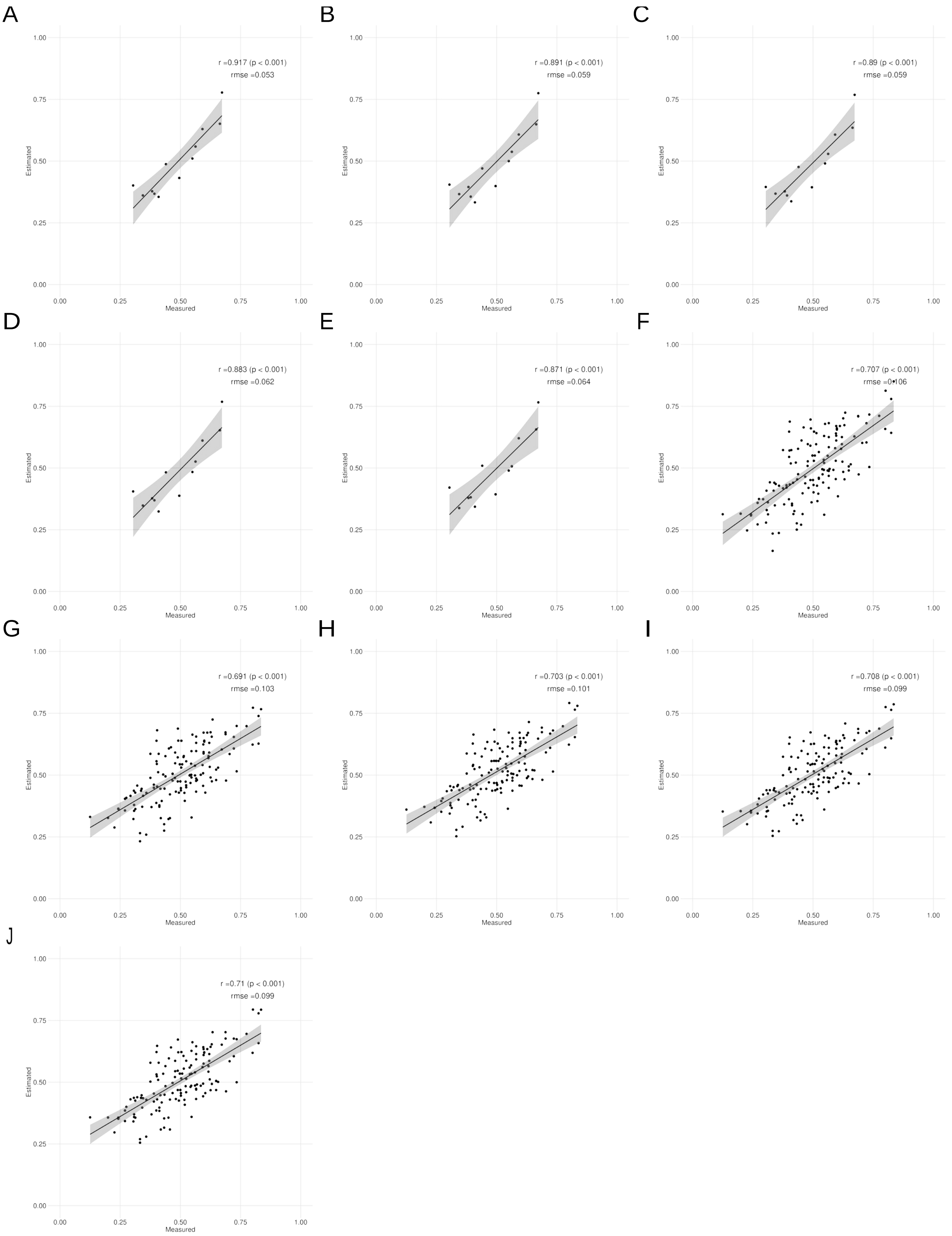
A
r =0.917 (p < 0.001)
rmse =0.053
B
r =0.891 (p < 0.001)
rmse =0.059
C
r =0.89 (p < 0.001)
rmse =0.059
D
r =0.883 (p < 0.001)
rmse =0.062
E
r =0.871 (p < 0.001)
rmse =0.064
F
r =0.707 (p < 0.001)
rmse =0.106
G
r =0.691 (p < 0.001)
rmse =0.103
H
r =0.703 (p < 0.001)
rmse =0.101
I
r =0.708 (p < 0.001)
rmse =0.099
J
r =0.71 (p < 0.001)
rmse =0.099
Estimated
Measured
0.00
0.25
0.50
0.75
1.00

Fig. S2.

Pearson's correlations of tested TI and TII DNAm reference matrix. **(A)** Pearson's correlation between measured (FT) proportions and estimated FT proportions using the 20 CpG reference matrix (n=12). **(B)** Pearson's correlation between measured (FT) proportions and estimated FT proportions using the 50 CpG reference matrix (n=12). **(C)** Pearson's correlation between measured (FT) proportions and estimated FT proportions using the 100 CpG reference matrix (n=12). **(D)** Pearson's correlation between measured (FT) proportions and estimated FT proportions using the 200 CpG reference matrix (n=12). **(E)** Pearson's correlation between measured (FT) proportions and estimated FT proportions using the 1000 CpG reference matrix (n=12). **(F)** Pearson's correlation between measured (FT) proportions and estimated FT proportions using the 10 CpG reference matrix in the validation dataset (n=174). **(G)** Pearson's correlation between measured (FT) proportions and estimated FT proportions using the 20 CpG reference matrix in the validation dataset (n=174). **(H)** Pearson's correlation between measured (FT) proportions and estimated FT proportions using the 50 CpG reference matrix in the validation dataset (n=174). **(I)** Pearson's correlation between measured (FT) proportions and estimated FT proportions using the 100 CpG reference matrix in the validation dataset (n=174). **(J)** Pearson's correlation between measured (FT) proportions and estimated FT proportions using the 200 CpG reference matrix in the validation dataset (n=174).

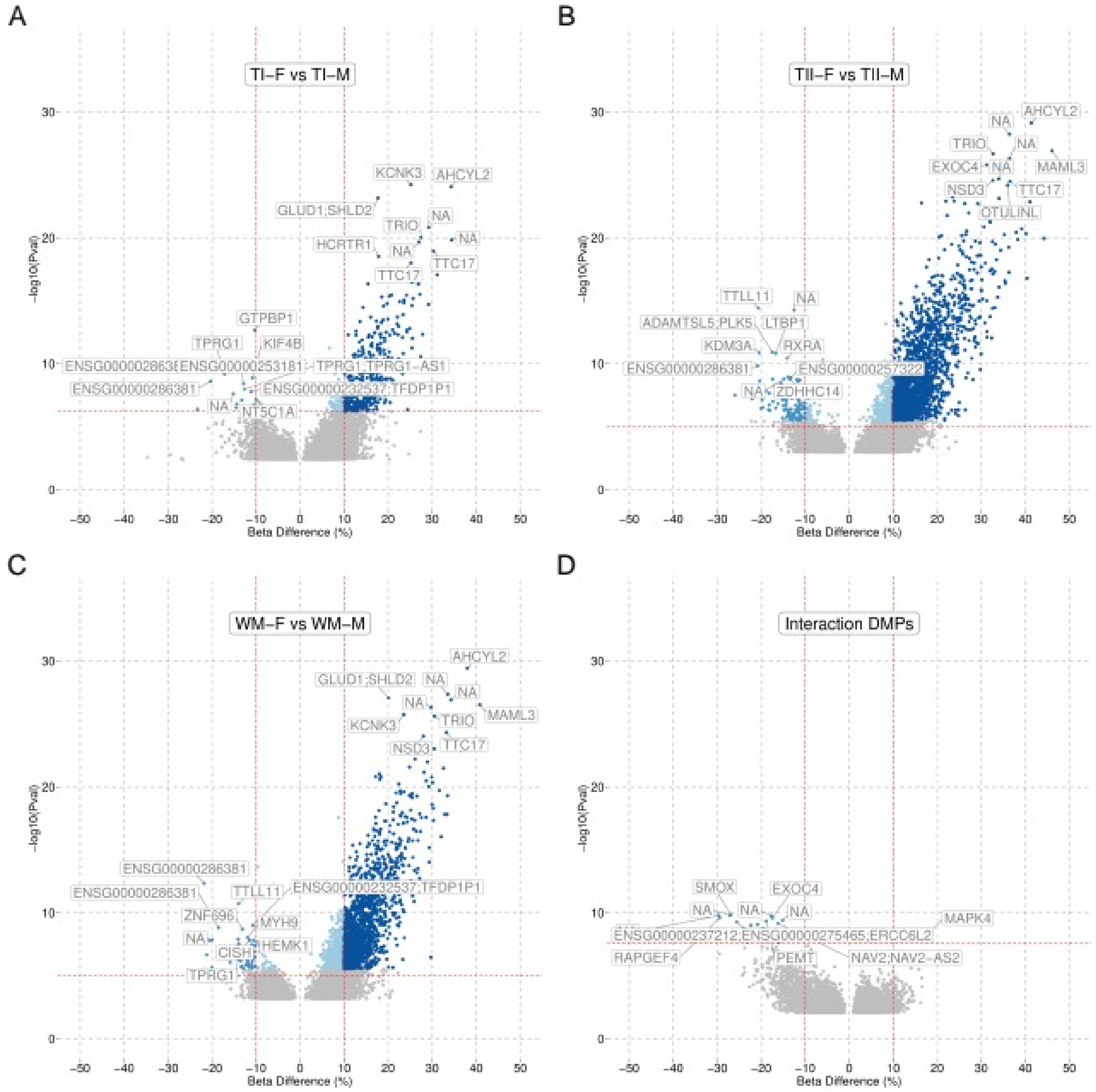

Fig. S3.

Sex analysis of TI, TII and WM DNAm samples. **(A)** Summary of the numbers of DMPs identified at differing Benjamini-Hochberg adjusted p-values in female vs males in type I (TI) fibres (n=5v7). **(B)** Summary of the numbers of DMPs identified at differing Benjamini-Hochberg adjusted p-values in female vs males in type TII (TII) fibres (n=5v7). **(C)** Volcano plot of DMPs identified between females and males in TI, with beta difference on the X-axis and -log10 p-value on the Y-axis. Royal blue: hypomethylated, dark blue: hypermethylated in TI vs TII fibres. The horizontal line represents the cutoff of the corresponding adjusted p-value <0.001 and the vertical lines represent the cutoff of the beta difference 10% and -10%. **(D)**Volcano plot of DMPs identified between females and males in TII fibres, with beta difference on the X-axis

and -log10 p-value on the Y-axis. Royal blue: hypomethylated, dark blue: hypermethylated in TI vs TII fibres. The horizontal line represents the cutoff of the corresponding adjusted p-value <0.001 and the vertical lines represent the cutoff of the beta difference 10% and -10%. **(E)** A summary of the numbers of DMPs identified at differing Benjamini-Hochberg adjusted p-values in female vs males in whole muscle (WM) (n=5v7). **(F)** Summary of the numbers of DMPs identified in an interaction model of females and males and TI and TII fibres (n=5v7). **(G)** Volcano plot of DMPs identified between females and males in WM, with beta difference on the X-axis and -log10 p-value on the Y-axis. Royal blue: hypomethylated, dark blue: hypermethylated in TI vs TII fibres. The horizontal line represents the cutoff of the corresponding adjusted p-value <0.001 and the vertical lines represent the cutoff of the beta difference 10% and -10%. **(H)** Volcano plot of DMPs identified between females and males in a fibre-type specific manner, with beta difference on the X-axis and -log10 p-value on the Y-axis. Royal blue: hypomethylated, dark blue: hypermethylated in TI vs TII fibres. The horizontal line represents the cutoff of the corresponding adjusted p-value <0.001 and the vertical lines represent the cutoff of the beta difference 10% and -10%.

Table S1.
Fibre pooling number per sample

Sample	TI	TII	TIIx
M1	179	87	1
M2	100	155	17
F1	102	147	11
F2	103	105	52
F3	110	140	19
M3	93	150	21
F4	158	109	8
M4	74	141	68
F5	174	88	17
M5	85	66	0
M6	110	90	41
M7	65	149	17
**Total Per FT**	1353	1427	272
**Total Fibres**	3052		
**Averages Per FT**	113	119	23

Table S2.
Myosin inclusion-list peptides used in DDA

m/z	z	NCE	Protein (UniProt)	Peptide sequence	Comment/ID
649.7726	2	28	MYH2 (Q9UKX2)	EQYEEEQESK	MYH2_Light#325460
831.3841	2	25	MYH2 (Q9UKX2)	QAEEAEEQSNTNLAK	271501
784.8811	2	28	MYH4 (Q9Y623)	LQDAEEHVEAVNSK	373262
510.2596	2	30	MYH7 (P12883)	SVNDLTSQR	512206
691.8311	2	28	MYH1 (P12882)	IQHELEEAEER	354524
838.3921	2	23	MYH1 (P12882)	QAEEAEEQSNVNLSK	179666
754.8886	2	25	MYH2 (Q9UKX2)	LQAAEEHVEAVNAK	258122
776.8836	2	28	MYH1 (P12882)	LQDAEEHVEAVNAK	373261
577.2776	2	20	MYH2 (Q9UKX2)	LQTESGEFSR	49982
380.5398	3	23	MYH1 (P12882)	SALAHALQSSR	186447
504.2211	2	23	MYH1 (P12882)	DEEIDQMK	103700
537.7701	2	20	MYH2 (Q9UKX2)	ANTEVAQWR	5960
495.2431	2	20	MYH4 (Q9Y623)	DEELDQLK	10399
729.8751	2	23	MYH7 (P12883)	IEDEQALGSQLQK	137294
501.7826	2	23	MYH2 (Q9UKX2)	EALVSQLSR	110011
750.8806	2	25	MYH7 (P12883)	LQEAEEAVEAVNAK	258253
1121.5181	2	28	MYH4 (Q9Y623)	ALQEAHQQTLDDLQMEEDK	299893
509.7801	2	20	MYH1 (P12882)	DTLVSQLSR	14589
505.9361	3	35	MYH4 (Q9Y623)	LLGSIEIDHTQYK	547045
597.8036	2	20	MYH2 (Q9UKX2)	EFEISNLQSK	17875
648.8306	2	28	MYH2 (Q9UKX2)	DTQIHLDDALR	315069
573.3061	2	20	MYH1 (P12882)	ALEDQLSEIK	4473
742.9011	2	28	MYH2 (Q9UKX2)	IEDEQALGIQLQK	349701
760.3691	2	25	MYH2 (Q9UKX2)	LAQESIMDIENEK	252888
618.6421	3	20	MYH4 (Q9Y623)	DLEESTLQHEATAAALR	12182
588.3111	2	23	MYH4 (Q9Y623)	TLEDQLSEIK	201077
749.9091	2	20	MYH4 (Q9Y623)	IEDEQALAIQLQK	36392
588.3111	2	23	MYH2 (Q9UKX2)	TLEDQLSELK	201078
1150.0436	2	25	MYH7 (P12883)	NDLQLQVQAEQDNLADAEER	264764
447.7501	2	20	MYH2 (Q9UKX2)	SLGTELFK	69306
831.4046	2	23	MYH4 (Q9Y623)	NAYEESLDHLETLK	170066
581.7961	2	20	MYH7 (P12883)	DFELNALNAR	10712
830.9546	2	20	MYH7 (P12883)	GQNVQQVIYATGALAK	33004
825.8981	2	28	MYH2 (Q9UKX2)	MEIDDLASNVETVSK	380973
767.4171	2	20	MYH1 (P12882)	AAYLQNLNSADLLK	1007
713.8611	2	20	MYH2 (Q9UKX2)	EAIFCIQYNIR	15918
1100.0296	2	25	MYH2 (Q9UKX2)	NDLQLQVQAEAEGLADAEER	264763
723.6741	3	23	MYH1 (P12882)	SELQAALEEAEASLEHEEGK	187941
974.4791	2	20	MYH7 (P12883)	NLQEEISDLTEQLGSSGK	58847
1168.5406	2	28	MYH2 (Q9UKX2)	LETDISQMQGEMEDILQEAR	365419

Table S3.
Participant characteristics

Participants		n=15
Sex	Male	8
	Female	7
Age	Median	34
	Min-Max	25-42
BMI	Mean (SD)	24.6 (3.0)
	Min-Max	18.0-28.7

Table S4.
Numbers of identified differentially expressed proteins between TI and TII fibres

	**TI**	**Vs**	**TII**
BH P-Value Threshold	Up		Down
BH P <0.001 LogFC >1 or <-1	110		34
BH P <0.001	283		55
BH P <0.005	363		62

Table S5.
Numbers of identified differentially methylated positions between TI and TII fibres

	**TI**	**Vs**	**TII**
BH P-Value Threshold	Up		Down
BH P <0.001 10% BetaDiff	8408		2780
BH P <0.001	47238		19811
BH P <0.005	61633		30374

Table S6.
Numbers of identified differentially methylated positions between female TI and male TI fibres

	**TI-F**	**Vs**	**TI-M**
BH P-Value Threshold	Up		Down
BH P <0.001 10% BetaDiff	417		17
BH P <0.001	474		25
BH P <0.005	695		62

Table S7.
Numbers of identified differentially methylated positions between female TII and male TII fibres

	**TII-F**	**Vs**	**TII-M**
BH P-Value Threshold	Up		Down
BH P <0.001 10% BetaDiff	1998		109
BH P <0.001	2459		151
BH P <0.005	3390		331

Table S8.
Numbers of identified differentially methylated positions between female WM and male WM fibres

	**WM-F**	**Vs**	**WM-M**
BH P-Value Threshold	Up		Down
BH P <0.001 10% BetaDiff	1510		49
BH P <0.001	2167		77
BH P <0.005	3224		231

Table S9.
Numbers of identified differentially methylated positions identified as interactions between sex and fibre type

		**Interaction**	
BH P-Value Threshold	Up		Down
BH P <0.001 10% BetaDiff	1		31
BH P <0.001	1		31
BH P <0.005	4		53
